# Risk factors for cancer among the working population in China: a cross-sectional analysis of baseline data from the WECAN project

**DOI:** 10.1136/bmjopen-2025-107063

**Published:** 2025-12-22

**Authors:** Kaige Sun, Baohua Wang, Feng J He, Puhong Zhang, Ning Wang, Yuan Li, Rong Luo, Changqiong Wang, Guangming Yi, Ting Yang, Lishu Zhou, Juan Zhou, Guohong Zhang, Jing Wu

**Affiliations:** 1 Beijing Physical Examination Center, Beijing, China; 2 National Center for Chronic and Non-Communicable Disease Control and Prevention, Chinese Center for Disease Control and Prevention, Beijing, China; 3 Wolfson Institute of Population Health, Barts and The London School of Medicine and Dentistry, Queen Mary University of London, London, UK; 4 Faculty of Medicine, University of New South Wales, The George Institute for Global Health, Sydney, New South Wales, Australia; 5 Nanchong Center for Disease Control and Prevention, Nanchong, Sichuan, China; 6 Xiangtan Center for Disease Control and Prevention, Xiangtan, Hunan, China; 7 Wuhai Center for Disease Control and Prevention, Wuhai, Inner Mongolia, China

**Keywords:** Risk Factors, Workplace, Prevalence, Cancer

## Abstract

**Objective:**

This study aims to assess the prevalence of cancer risk factors in China’s working population and provide evidence for formulating more targeted workplace cancer prevention strategies.

**Design:**

A cross-sectional study utilising the baseline data from the Comprehensive Workplace Intervention for Cancer Prevention in China (WECAN) project, a stepped-wedge cluster randomised controlled trial.

**Setting:**

15 workplaces from three cities in China: Wuhai (Northern China), Nanchong (Western China) and Xiangtan (Southern China).

**Participants:**

A total of 841 participants (56 employees per workplace) were recruited through stratified sampling based on sex and work type.

**Primary outcome measures:**

The prevalence of self-reported cancer risk factors, including smoking, alcohol consumption, physical inactivity, obesity, unhealthy diet, betel quid chewing, exposure to harmful gases or substances, biological screening and vaccination.

**Results:**

The study included 841 participants (mean age 40.5±8.9 years; 61.4% male, 57.4% blue-collar workers, 47.3% aged <40 years), with 36.4% reporting family cancer history. The reported cancer risks included smoking (36.9%, male–female OR=40.4 (95% CI 21.6 to 75.7)), alcohol consumption (63.6%, male–female OR=4.3 (95% CI 3.0 to 6.0)), physical inactivity (49.1%), central obesity (36.7%, male–female OR=3.2 (95% CI 2.3 to 4.5)), unhealthy diet (95.8% with two or more of six unhealthy dietary habits), betel quid chewing (18.8%, male–female OR=3.3 (95% CI 1.9 to 5.9), with 97.5% in Xiangtan), preference for hot foods (5.0%, older–younger OR=2.5 (95% CI 1.1 to 5.6)), household air pollution (7.0%, higher risk among lower-educated individuals), unprotected occupational exposure (6.1%, male–female OR=2.8 (95% CI 1.3 to 6.0), blue-white collar OR=2.1 (95% CI 1.0 to 4.5)), no screening for hepatitis B surface antigen (46.4%) and helicobacter pylori (54.9%), and unvaccinated against hepatitis B virus (28.1%) and human papillomavirus (63.5% among females≤45 years). Regional heterogeneity was observed for nearly all risk factors.

**Conclusions:**

The working population faces significant cancer risk factors, with variations across populations and regions highlighting the need for targeted interventions.

**Trial registration number:**

ChiCTR2200058680.

**STRENGTHS AND LIMITATIONS OF THIS STUDY**

- The study covered diverse workplaces across multiple regions in China, boosting the geographical and occupational representativeness of the findings.
- The data were collected by trained professionals face-to-face through a customised electronic system, effectively ensuring the data quality and enabling comparability among different sites.
- The sample size was designed to detect changes in Healthy Lifestyle Index Score (HLIS; the primary outcome of the WECAN study), which may limit the power to robustly estimate cancer risk factor prevalences, especially those with low prevalences.
- The sampling was not nationally representative, which may restrict generalisability to the broader working population.
- Certain lifestyle factors and exposures relied on self-reported data and may be subject to recall, reporting and social desirability biases.

## Introduction

 Cancer is the second leading cause of death in the world. It was estimated that there would be 20 million new cancer patients and 9.7 million deaths worldwide in 2022, including 4.8 million new cancer patients and 2.6 million deaths in China. Projections show that by 2050, there will be more than 35 million new cancer patients worldwide, an increase of 77% from 2022. The rapid increase in the global cancer burden is not only related to population ageing and growth, but also reflects changes in people’s exposure to cancer risk factors, with smoking, alcohol consumption and obesity being the main risk factors leading to an increase in cancer prevalence.^1^

Specifically, smoking is linked to at least 20 different types or subtypes of cancer (such as respiratory system, urinary system, head and neck cancer, etc) and about 2.4 million people die of tobacco-related cancer every year.^2^ Alcohol consumption increases the risk of cancers of the oral cavity, oropharynx, oesophagus (squamous cell carcinoma), colon, rectum, liver and intra-hepatic bile duct, larynx and female breast. In 2016, alcohol consumption caused about 376 000 cancer deaths, accounting for 4.2% of all cancer deaths.^2^ There is strong evidence that physical activity reduces the risk of bladder, breast, colorectal and other cancers by about 10%–20%, and the effect increases with increasing activity levels.^2^ Considerable epidemiological evidence supports an association between overweight and obesity and cancer risk. Obesity in adulthood significantly increases the risk of more than 10 types of cancer, including postmenopausal breast cancer and colorectal cancer.^3 4^ Unhealthy dietary habits (low intake of vegetables, fruits and dietary fibre, consumption of too much red meat and processed meat, high-salt diet, etc) and preference for hot foods are closely related to the occurrence of colorectal cancer, gastric cancer and oesophageal cancer.^5^,^7^ Betel quid chewing is associated with the development of oral diseases including oral submucous fibrosis, oral leucoplakia and oral cancer.^8 9^

In addition to the common lifestyle risk factors mentioned above, infections and occupational exposure to harmful substances are also important risk factors for cancer. It is estimated that about one in eight new cancer cases (220 million cases) in 2018 was caused by infections, with *Helicobacter pylori* (HP) causing about 810 000 new cases of gastric cancer, chronic hepatitis B virus (HBV) infection causing about 360 000 cases of hepatocellular carcinoma and human papillomavirus (HPV) causing all cervical cancer cases worldwide (570 000 cases) and some reproductive tract and oropharyngeal cancer cases (120 000 cases in total).^2^ The WHO’s Global Burden of Disease Study 2017 estimates that about 334 000 cancer deaths can be attributed to occupational exposure, with asbestos, silica and diesel exhaust being the main hazardous substances.^10^ Additionally, cooking-related household air pollution exposure increases the risk of lung cancer.^11 12^

Moreover, cancer risk rises rapidly after the age of 40, and this age group primarily comprises working population who play key roles in their families, organisations and society. However, epidemiological evidence on cancer risk factors among working adults remains limited both globally and in China. Most existing studies have focused on the general population or on specific occupational exposures (such as asbestos, silica dust, diesel exhaust in particular industries) rather than providing a comprehensive assessment across the broader working population.^13 14^ National burden studies in China have quantified cancer risks attributable to occupational or lifestyle factors, but they have not described the overall distribution of these risk factors among employed adults.^15^,^17^ This knowledge gap limits the understanding of cancer prevention priorities in workplaces and hinders the development of tailored, workplace prevention strategies.

The Comprehensive Workplace Intervention for Cancer Prevention in China (WECAN) project is a cluster randomised controlled trial (cRCT) designed to evaluate the effectiveness of a cancer prevention programme implemented at the workplace.^18^ Leveraging WECAN’s baseline data, this study aims to systematically assess the prevalence and distribution of cancer risk factors among China’s working population, to fill an important evidence gap and to provide timely evidence for formulating more targeted workplace cancer prevention strategies.

## Methods

### Study design, setting and participants

This is a cross-sectional study utilising the baseline data from the WECAN project and reported following Strengthening the Reporting of Observational studies in Epidemiology (STROBE) guidelines. WECAN is a stepped-wedge cRCT involving 15 workplaces, with 5 workplaces entering the intervention at each 6-month step and all randomly selected participants being followed up every 6 months over a total period of 2.5 years for effectiveness evaluation. Detailed protocol has been published elsewhere.^18^ The 15 workplaces were selected from Northern China (Wuhai City, Inner Mongolia Autonomous Region), Western China (Nanchong City, Sichuan Province) and Southern China (Xiangtan City, Hunan Province), covering three sectors: energy and chemical industry (eight workplaces), steel manufacturing (four workplaces) and public utilities and environmental protection (three workplaces).

Inclusion criteria for workplaces were as follows: (1) at least 100 full-time employees; (2) an average turnover rate <20% in the past 3 years; (3) willingness of the employer to participate and support project implementation and (4) routine provision of employee health examinations. To ensure coverage of both white-collar (office-based) and blue-collar (production-based) workers, workplaces with a mix of physically demanding and non-physical occupations—such as those in the manufacturing sector—were prioritised. No additional exclusion criteria were applied. Workplaces were recruited through local Centres for Disease Control and Prevention (CDCs), which recommended eligible workplaces to the project team. The team subsequently conducted preliminary screening through onsite or remote assessments.

In each workplace, 56 employees meeting the criteria were randomly selected for the baseline and follow-up survey through stratified sampling based on sex and work type (white-collar vs blue-collar), comprising a total of 841 participants.

- Inclusion criteria: employees agreed to receive the intervention through smartphone applications, were capable of ensuring regular work attendance, had no intention of retiring or quitting the job within the next 3 years, consented to be surveyed regarding the outcome assessment and signed the informed consent form.
- Exclusion criteria: employees who had been diagnosed with cancer, were pregnant or preparing for pregnancy, or had other physical, psychological or social impediments preventing them from participating in the assessment survey.

### Definitions of key variables

- Smoking was defined as current smoking, referring to individuals who had smoked continuously or cumulatively for at least 6 months.
- Alcohol consumption was defined as alcohol intake in the past 6 months.
- Physical inactivity was characterised as engaging in less than 150 min of moderate-intensity physical activity or less than 75 min of high-intensity physical activity per week.^19^
- Overweight was defined as a Body Mass Index (BMI) between 24 and 28 kg/m^²^, obesity as a BMI≥28 kg/m^²^ and central obesity as a waist circumference of ≥90 cm for men and ≥85 cm for women.^20^
- Dietary patterns were assessed according to the Chinese Dietary Guidelines (2022),^21^ which identified six types of unhealthy dietary habits: insufficient vegetable intake (<300 g/day), insufficient fruit intake (<200 g/day), low dietary fibre intake (<25 g/day), excessive red meat consumption (>500 g/week), high-salt diet (moderate to salty taste preferences, or the consumption of takeout meals, processed meats, pickled vegetables, fermented bean curd or salty snacks at least once per week) and frequent consumption of processed meat (consumption of processed meat at least once per week).
- Preference for hot foods referred to people who prefer consuming foods that are served at a high temperature.
- Cooking-related household air pollution exposure was assessed based on cooking habits and range hood usage, referring to individuals who cook with high heat, like stir-frying or deep frying, but do not frequently use a range hood.
- Family history of cancer was defined as having at least one first-degree relative (parents, full siblings or children) or second-degree relative (grandparents, uncles or aunts) who had ever been diagnosed with any of the specified cancers.

### Data collection

The questionnaire gathered information on employees’ demographics, lifestyle factors (smoking, alcohol consumption, physical activity and diet), exposure history to harmful gases/substances, personal medical history, family history and biological test results. Physical measurements included height, weight and waist circumference. Lifestyle behaviours such as alcohol consumption, physical activity and dietary habits were assessed based on participants’ behaviours over the past 6 months, while biological test results were obtained from routine health examination reports.

The questionnaire was developed following the framework of the Healthy Lifestyle Index Score (HLIS),^22^ with each component adapted from well-validated instruments widely used in previous studies.^23 24^ To ensure local relevance, core items were adapted from the questionnaires used in the China Chronic Disease and Risk Factor Surveillance (CCDRFS) and the Cancer Screening Programme in Urban China (CanSPUC),^25 26^ both of which have been extensively validated and implemented at the national level. A pilot survey before the main study was conducted to assess clarity and feasibility, and minor adjustments were made accordingly. The full questionnaire has been included as an online supplemental material (Baseline Assessment Tool for WECAN project).

All questionnaires and physical assessments were conducted one-on-one by trained staff from the CDCs across the three cities. To reduce potential biases, several measures were adopted in this study. Surveyors received standardised training in data collection and interview techniques to ensure consistency and minimise measurement errors. Interviews were conducted in private settings to protect confidentiality and enhance the reliability of self-reported information. Quality control was implemented through the mobile Electronic Data Capture system, which included audio recording and logical verification functions to ensure the standardisation and accuracy of survey data. In addition, body size was measured onsite, and data from routine health examination reports were referenced to improve measurement accuracy.

The baseline survey was conducted from April to July 2023.

### Statistical analysis

Continuous variables were described by mean±SD, and categorical variables were described by frequency and proportion (%). The χ^2^ test (for categorical variables) and t-test or analysis of variance (for continuous variables) were used for univariate analysis to compare the risk factor exposure levels among different populations. For each cancer risk factor (eg, smoking, alcohol consumption), a separate multivariable logistic regression model was performed to estimate the ORs across different populations, with all covariates simultaneously adjusted for each other in the model. Covariates included sex (female as reference), work type (white-collar as reference), age group (<40 years as reference), education level (junior high school or below as reference), marital status (married/cohabiting as reference), family history of cancer (no history as reference) and city (Nanchong as reference). To account for multiple comparisons across several cancer risk factors, p-values were adjusted using the Benjamini–Hochberg false discovery rate procedure. A two-sided p value<0.05 was considered statistically significant. Standardisation of the rates of these risk factors was performed using the age-specific and sex-specific distribution of the employed population from China’s seventh National Population Census as the standard population. Statistical analyses were performed using SAS V.9.4.

### Patient and public involvement

Representatives from the study cities and workplaces were involved in several key aspects of the study. They assisted in recruiting representative employees by introducing sampling methods and understanding the distribution of employees by sex and work type. They also contributed to the development of the questionnaire, ensuring that the questions were clear, culturally appropriate and covered major cancer risk factors. Additionally, they participated in a pilot survey using the electronic data collection system to test its accuracy, feasibility and acceptability, make necessary adjustments and identify training needs.

## Results

The study included a total of 841 participants, with a mean age of 40.5±8.9 years. Among them, 516 (61.4%) were men, 483 (57.4%) were blue-collars and 398 (47.3%) were under 40 years old. Additionally, 686 (81.6%) participants were married or cohabiting, and 306 (36.4%) had a family history of cancer. The educational distribution was as follows: 13.2% had completed junior high school or below, 19.0% had attended high school or vocational school or technical school, 31.2% had a junior college education and 36.6% had a bachelor’s degree or above (table 1).

**Table 1:** Baseline characteristics of the participants

Characteristics	N (proportion, %)
**Total**	841 (100)
Sex	
Male	516 (61.4)
Female	325 (38.6)
Work type	
White-collar	358 (42.6)
Blue-collar	483 (57.4)
Age (years)	
<40	398 (47.3)
≥40	443 (52.7)
Education	
Junior high school or below	111 (13.2)
High school/vocational school/technical school	160 (19.0)
Junior college	262 (31.2)
Bachelor’s degree or above	308 (36.6)
Marital status	
Married/cohabiting	686 (81.6)
Unmarried/divorced/widowed	155 (18.4)
Family history of cancer	
Yes	306 (36.4)
No	535 (63.6)
City	
Wuhai	280 (33.3)
Nanchong	281 (33.4)
Xiangtan	280 (33.3)

The smoking rate in the total population was 36.9%, and the alcohol consumption rate was 63.6%. Physical inactivity was observed in 49.1% of individuals. The mean BMI of the study population was 25.4±3.9 kg/m^²^, while the obesity and central obesity rates were 23.9% and 36.7%, respectively. Unhealthy dietary habits were identified, including insufficient vegetable intake (59.3%), insufficient fruit intake (75.3%), low dietary fibre intake (58.5%), high-salt diet (95.6%), excessive red meat consumption (64.7%) and frequent processed meat consumption (22.5%); 95.8% of people have two or more types of unhealthy dietary habits. Additionally, 18.8% chewed betel quid and 5.0% of participants preferred hot foods. Cooking-related household air pollution exposure was reported by 7.0% of the population. Furthermore, 6.1% of individuals had a history of occupational exposure to harmful substances without protection for more than 1 year. In terms of infections, 53.6% of individuals had been ever tested for hepatitis B surface antigen (HBsAg), and 45.1% for HP, with positive rates of 7.3% and 22.7% among those tested, respectively. Regarding vaccination status, 28.1% of the total population had not received the hepatitis B vaccinations, while 63.5% of women under 45 had not been vaccinated against HPV (table 2).

**Table 2:** Proportions of cancer risk factors in the study population

Cancer risk factor	N (proportion, %)
**Total**	**841** (**100.0**)
Smoking*	310 (36.9)
Alcohol consumption	535 (63.6)
Physical inactivity†	413 (49.1)
BMI‡	
Normal	334 (39.7)
Overweight	306 (36.4)
Obesity	201 (23.9)
Central obesity§	309 (36.7)
Unhealthy diet¶	806 (95.8)
Insufficient vegetable intake	499 (59.3)
Insufficient fruit intake	633 (75.3)
Low dietary fibre intake	492 (58.5)
High-salt diet	804 (95.6)
Excessive red meat consumption	544 (64.7)
Frequent consumption of processed meat	189 (22.5)
Preference for hot foods**	42 (5.0)
Betel quid chewing	158 (18.8)
Cooking-related household air pollution exposure††	59 (7.0)
Unprotected occupational exposure (>1 year**)**	51 (6.1)
Among those who have exposure history	
Diesel exhaust, coal smoke, coal ash, etc	20 (39.2)
Asbestos	4 (7.8)
Other exposures^‡‡^	30 (58.8)
HBsAg testing rate	451 (53.6)
Among tested, positivity rate	33 (7.3)
HP testing rate	379 (45.1)
Among tested, positivity rate	86 (22.7)
Unvaccinated against HBV	236 (28.1)
Unvaccinated against HPV among females aged ≤45 years	148 (63.5)

* Smoking: current smoking.

† Physical inactivity: <150 min of moderate-intensity or <75 min of high-intensity physical activity per week.

‡ BMI: classified as normal (<24.0 kg/m²), overweight (24.0–27.9 kg/m²), obesity (≥28.0 kg/m²).

§ Central obesity: waist circumference ≥90 cm for men and ≥85 cm for women.

¶ Unhealthy diet: meeting ≥2 of the following: insufficient vegetable intake (<300 g/day), insufficient fruit intake (<200 g/day), low dietary fiber intake (<25 g/day), excessive red meat consumption (>500 g/week), high-salt diet (moderate to salty taste preferences, or the consumption of takeout meals, processed meats, pickled vegetables, fermented bean curd or salty snacks at least once per week) and frequent consumption of processed meat (consumption of processed meat at least once per week).

** Preference for hot foods: frequent consumption of foods served at high temperatures.

†† Cooking-related household air pollution exposure: cooking with high heat (eg, stir-frying or deepfrying) without frequent use of a range hood.

‡‡ Other exposures: beryllium, uranium, radon, chromium, cadmium, nickel, silica, etc.

HPV, human papillomavirus.BMI, Body Mass Index; HBsAg, hepatitis B surface antigen; HBV, hepatitis B virus; HP, Helicobacter pylori.

Multivariable analysis uncovered notable disparities in cancer risk factors across different demographic groups and cities.

Males had significantly higher prevalence of smoking, alcohol consumption, obesity, central obesity and unhealthy dietary habits (including insufficient fruit, low fibre, high-salt, excessive red-meat intake and frequent consumption of processed meat) than females. Moreover, they were more likely to chew betel quid, had a larger proportion of individuals who had never undergone HP testing and a higher rate of unprotected occupational exposure to hazards (table 3, online supplemental table S1).

**Table 3:** Associations between participant characteristics and specific cancer risk factors: multivariable-adjusted ORs (OR (95% CI))^*^

Characteristics	Cancer risk factors							
	Smoking	Alcohol consumption	Physical inactivity	Obesity	Central obesity	Unhealthy diet	Preference for hot foods	Betel quid chewing
Sex (male vs female)	40.4 (21.6–75.7)^†^	4.3 (3.0–6.0)^†^	0.9 (0.7–1.2)	2.1 (1.5–3.1)^†^	3.2 (2.3–4.5)^†^	4.4 (2.0–9.8)^†^	0.8 (0.4–1.5)	3.3 (1.9–5.9)^†^
Work type (Blue-collar vs white-collar)	0.9 (0.6–1.3)	1.0 (0.7–1.4)	1.1 (0.8–1.5)	0.8 (0.5–1.1)	1.0 (0.7–1.3)	0.8 (0.4–1.7)	1.4 (0.7–2.9)	1.3 (0.7–2.2)
Age group (≥40 vs <40)	0.9 (0.6–1.4)	0.9 (0.6–1.3)	0.9 (0.6–1.2)	1.0 (0.7–1.5)	1.2 (0.9–1.7)	0.6 (0.3–1.4)	2.5 (1.1–5.6)	0.6 (0.4–1.1)
Education (vs junior high school or below)								
High school^‡^	0.7 (0.4–1.3)	1.6 (0.9–2.9)	0.8 (0.5–1.4)	0.9 (0.5–1.6)	0.8 (0.4–1.3)	1.3 (0.3–6.9)	0.7 (0.3–1.9)	3.5 (0.9–13.7)
Junior college	0.6 (0.3–1.1)	2.4 (1.4–4.1)^†^	0.9 (0.5–1.4)	0.7 (0.4–1.3)	0.6 (0.4–1.0)	0.3 (0.1–1.4)	0.9 (0.4–2.3)	1.7 (0.5–6.1)
Bachelor's degree or above	0.2 (0.1–0.4)^†^	2.0 (1.1–3.6)	0.9 (0.5–1.6)	0.6 (0.3–1.2)	0.5 (0.3–1.0)	0.4 (0.1–2.0)	0.7 (0.2–2.2)	0.7 (0.2–2.7)
Marital status (unmarried/divorced/widowed vs married/cohabiting)	0.7 (0.4–1.1)	1.0 (0.6–1.5)	1.2 (0.8–1.8)	1.1 (0.7–1.7)	0.8 (0.6–1.3)	2.9 (0.7–13.1)	0.7 (0.2–2.3)	0.8 (0.4–1.6)
Family history of cancer (yes vs no)	1.0 (0.7–1.5)	0.8 (0.6–1.1)	1.3 (0.9–1.7)	1.0 (0.7–1.5)	1.3 (0.9–1.8)	0.5 (0.2–1.1)	0.8 (0.4–1.7)	0.9 (0.5–1.5)
City (vs Nanchong)								
Wuhai	0.5 (0.3–0.9) ^†^	0.2 (0.1–0.3)^†^	1.3 (0.8–1.9)	3.0 (1.9–4.9)^†^	3.0 (1.9–4.7)^†^	0.9 (0.4–2.5)	1.8 (0.8–4.4)	0.3 (0.0–2.5)
Xiangtan	0.6 (0.4–1.0)	0.4 (0.3–0.6)^†^	0.6 (0.4–0.9)^†^	1.3 (0.8–2.1)	1.9 (1.3–2.8)^†^	2.9 (1.1–7.7)	0.5 (0.2–1.4)	147.1 (44.2–489.6)^†^

Characteristics	Cancer risk factors					
	Cooking-related household air pollution	Unprotected occupational exposure (>1 year)	Never-tested for HBsAg	Never-tested for HP	Unvaccinated against HBV	Unvaccinated against HPV among females aged ≤45 years
Sex (males vs females)	1.1 (0.6–2.1)	2.8 (1.3–6.0)^†^	1.3 (1.0–1.8)	1.4 (1.0–2.0)	1.3 (0.9–1.8)	–
Work type (blue-collar vs white-collar)	1.2 (0.6–2.1)	2.1 (1.0–4.5)	1.4 (1.0–1.9)	1.1 (0.7–1.5)	1.5 (1.0–2.1)	1.4 (0.8–2.7)
Age group (≥40 vs <40)	0.8 (0.4–1.6)	0.6 (0.3–1.2)	0.9 (0.6–1.3)	0.6 (0.4–0.9)	1.5 (1.0–2.2)	2.2 (1.1–4.5)
Education (vs junior high school or below)						
High school^‡^	0.4 (0.1–0.8)	2.8 (0.6–13.3)	0.5 (0.3–0.8)	0.4 (0.2–0.8)	0.8 (0.5–1.3)	3.1 (0.8–12.4)
Junior college	0.3 (0.2–0.8)^†^	1.1 (0.2–5.6)	0.3 (0.2–0.5)^†^	0.2 (0.1–0.5)^†^	0.9 (0.5–1.5)	5.9 (1.6–21.2)^†^
Bachelor's degree or above	0.4 (0.1–0.9)	1.2 (0.2–6.0)	0.3 (0.1–0.5)^†^	0.2 (0.1–0.4)^†^	0.6 (0.3–1.1)	3.6 (1.0–13.1)
Marital status	1.5 (0.7–3.0)	0.5 (0.2–1.3)	1.3 (0.9–2.0)	1.5 (1.0–2.5)	0.8 (0.5–1.2)	0.4 (0.2–0.9)
(Unmarried/divorced/widowed vs married/cohabiting)						
Family history of cancer (yes vs no)	1.4 (0.8–2.5)	1.4 (0.8–2.7)	1.0 (0.7–1.3)	1.0 (0.7–1.5)	1.0 (0.7–1.5)	0.6 (0.3–1.1)
City (vs Nanchong)						
Wuhai	1.2 (0.6–2.6)	0.0 (0.0–0.3)^†^	2.3 (1.5–3.5)^†^	9.1 (5.7–14.4)^†^	2.3 (1.5–3.5)^†^	2.0 (0.8–4.9)
Xiangtan	0.4 (0.2–0.9)	1.0 (0.5–1.8)	1.5 (1.0–2.1)	9.1 (6.1–13.7)^†^	0.8 (0.5–1.2)	1.1 (0.5–2.1)

* Multivariable logistic regression model was performed for each cancer risk factor, with covariates including sex, work type, age group, education, marital status, family history of cancer and city. All covariates were simultaneously adjusted for each other.

† Adjusted p value<0.05.

‡ High school included vocational school and technical school.

HBsAg, hepatitis B surface antigen; HBV, hepatitis B virus; HPV, human papillomavirus.

Compared with office workers, workshop employees had lower HBsAg testing coverage and hepatitis B vaccination rates. Additionally, they exhibited a higher prevalence of unprotected occupational exposure (table 3).

Older employees were more likely to consume hot-temperature food and less likely to be vaccinated against hepatitis B or HPV. On the contrary, younger employees reported a higher rate of high-salt diet and a greater proportion of those who had never undergone HP testing (table 3, online supplemental table S1).

Individuals with higher education generally had lower rates of smoking and central obesity. However, they demonstrated a higher rate of alcohol consumption. They were also less exposed to cooking-related household air pollution and had a lower proportion of people who had never undergone HBsAg and HP tests, yet a higher rate of people who had not been vaccinated against HPV (table 3).

Statistical differences were absent for almost all risk factors across marital statuses and cancer family history, except that the HPV vaccination rate varied significantly by marital status. (table 3).

Almost all risk factors—except for preference for hot foods, insufficient vegetable intake, high-salt diet and HPV vaccination—showed regional differences across the three cities. Compared with Nanchong, both Wuhai and Xiangtan had lower smoking and alcohol consumption rates but higher central obesity rates. A greater proportion of the participants in Xiangtan met physical activity standards. In diet, Wuhai had a higher rate of insufficient fruit and dietary fibre intake, yet a lower proportion of excessive red meat. In Xiangtan, the rates of insufficient fruit intake and frequent consumption of processed meat were higher than Nanchong, and its betel quid chewing rate was much higher than that of Wuhai and Nanchong. Regarding occupational exposure to hazardous substances, Wuhai was better than Nanchong, but it had a higher proportion of people who had never undergone HBsAg and HP tests or received the hepatitis B vaccine. Likewise, Xiangtan also had a higher proportion of people in such situations compared to Nanchong (table 3, online supplemental table S1).

Furthermore, univariable analyses also revealed additional differences in cancer risk factors across various populations, as detailed in the online supplemental table S2. In addition, standardization of the prevalence of these risk factors was performed to improve comparability, as detailed in the online supplemental table S3.

## Discussion

Overall, the exposure levels of cancer risk factors in this working population are concerning. High prevalence of smoking, alcohol consumption, overweight/obesity, physical inactivity and unhealthy diet, together with relatively low screening rates for HP and HBsAg (around or below 50%), indicates a substantial burden of modifiable risks. These patterns suggest that such risks may be influenced by workplace norms and daily routines, with male-dominated industries potentially fostering unhealthy behaviours. In addition, the distribution of major risk factors varied by different characteristics and regions, suggesting the need for targeted intervention strategies.

To enhance comparability, the reported rates in the following comparisons were all standardised. Overall, our findings indicate that participants in this working population exhibited higher rates of several lifestyle-related and obesity-related risk factors compared with both previous occupational cohort studies and the general adult population in China (details in online supplemental table S4).^27^,^30^ These differences may be attributable to a combination of factors, including the predominance of male employees, specific occupational characteristics and social or workplace environments that may promote unhealthy behaviours. Variations in survey timing, regional culture, economic conditions and health awareness across studies may further contribute to the observed differences. Importantly, the elevated risk factor levels observed among the working population in industrial sectors suggest that they may represent an under-recognised priority population for cancer prevention and chronic disease control in China, highlighting the need for workplace health interventions tailored to the characteristics of the working population, focusing on reducing smoking and alcohol use, promoting physical activity, managing body weight and supporting healthy behaviours in the working population.

Although infections and occupational hazards remain major contributors to cancer in China, our findings reveal substantial gaps in prevention among this working population. Approximately half of the participants had never been tested for HBsAg or HP, 28.1% reported no history of hepatitis B vaccination and 63.5% of women under 45 had not received HPV vaccination. These low uptake levels likely reflect limited awareness of infection-related cancer risks, insufficient workplace screening arrangements and competing work demands that constrain opportunities to seek preventive care. In parallel, 5.9% of participants reported unprotected exposure to hazardous substances for over 1 year, including asbestos (7.8%), diesel exhaust (39.2%) and other harmful substances (58.8%), indicating persistent gaps in occupational safety. Such exposure patterns may stem from inadequate safety management systems, inconsistent enforcement of protective regulations or insufficient provision and use of personal protective equipment. Taken together, these findings underscore the need to strengthen health education, expand workplace screening and vaccination programmes and improve hazard monitoring and engineering controls to reduce infection-related and occupation-related cancer risks among working population.

This study also reveals significant clustering of risk factors within certain populations, highlighting important targets for workplace cancer prevention. Men consistently showed higher exposure levels across lifestyle-related, biological and occupational factors, which may partly explain their higher incidence and mortality of several chronic diseases and cancers. These patterns likely reflect sex-related differences in occupational roles, health behaviours and risk awareness. Men are more often engaged in physically demanding or hazardous work and may be less likely to adopt protective lifestyles or seek preventive care. These findings highlight the need for sex-specific prevention strategies, particularly early and sustained interventions targeting men during their working years, to reduce long-term health risks and narrow sex disparities in health outcomes. This persistent imbalance may partially explain shorter life expectancy in men compared with women. In addition, employees with lower educational levels exhibited higher rates of smoking, central obesity, lack of biological screening and exposure to cooking-related household air pollution. These findings indicate that workplace health promotion should particularly prioritise individuals with lower educational attainment through accessible and practical measures, such as plain-language health education, onsite screening and counselling, and incentive-based lifestyle programmes.

Furthermore, the disparities in risk factor levels across the three cities indicate that workplace health interventions must be region-specific. In Xiangtan, the high prevalence of betel quid chewing (55.0%) reflects longstanding cultural practices; it was even inscribed in 2016 on Hunan Province’s fourth batch of intangible cultural heritage, emphasising its social significance. In Nanchong, elevated smoking (40.6%) and alcohol consumption (79.7%) are likely influenced by local cultural norms, as Sichuan Province produces nearly 40% of China’s distilled spirits and ranks among the top five provinces in tobacco production, reflecting a pervasive ‘smoking-and-drinking culture’. In Wuhai, the substantial burden of central obesity (50.0%) may be related to lifestyle and metabolic patterns common in colder climates, such as reduced outdoor physical activity and greater consumption of energy-dense foods; the predominance of small private enterprises and relatively lower economic levels may further limit employee welfare and health awareness, contributing to low screening rates for infection markers (37.5% for HBsAg and 24.6% for HP). Tailoring interventions to these local contexts—for example, introducing betel quid cessation or harmless treatment policy in Xiangtan, tobacco and alcohol reduction programmes in Nanchong, and obesity management and enhanced infection screening in Wuhai—will be crucial for achieving meaningful improvements in workplace health and cancer prevention.

The WECAN study was originally designed as an intervention trial, using HLIS as the primary outcome. The overall study design and sample size calculation were based on evaluating changes in HLIS before and after the intervention. However, HLIS was not described or analysed in the present study for the following reasons. First, HLIS is a composite indicator suitable for assessing overall lifestyle improvements at the individual or group level. Its abstract scoring does not directly reflect the prevalence of specific cancer risk factors in the population and is less useful for informing targeted prevention strategies. Second, baseline HLIS scores will be reported in detail in the main WECAN study publication.

Cautions should be made when extrapolating the national prevalences of those risk factors. Participants were recruited from 15 workplaces across three cities, covering the energy and chemical industry, steel manufacturing and public utilities and environmental protection sectors to include diverse occupational environments. The sampling framework was based on workplace recommendations from local CDCs followed by feasibility assessments, rather than probability-based sampling. As such, the participating workplaces reflect characteristics of the recruited workplaces rather than forming a nationally representative sample of all Chinese workplaces. In addition, the sample size calculated to evaluate HLIS change between groups may have limited power for robust estimation of cancer risk factor prevalences, especially for those with low prevalences.

Finally, although several measures were implemented during data collection, self-reported variables may still be affected by recall and reporting biases, including social desirability bias. Specifically, participants may under-report unhealthy behaviours, such as smoking, alcohol consumption, certain disease history and family history of cancer, and over-report healthy behaviours, such as fruit and vegetable intake and physical activity. These biases may lead to underestimation or overestimation of the true prevalence of the investigated lifestyle and health-related risk factors.

### Strengths and limitations

First, our study covered diverse workplace settings across multiple geographic regions in China, enhancing the geographical and occupational representativeness of the findings. Second, the data were collected through rigorously designed assessment tools and standardised procedures, ensuring high data quality and comparability across sites. However, some limitations should be noted. First, the sample size was calculated to detect HLIS changes (the primary outcome of the WECAN study), which may have limited power for robust estimation of cancer risk factor prevalences, especially those with low prevalence. Second, although participants were recruited from multiple workplaces, the sampling was not nationally representative, which may limit the generalisability of the findings. Third, several lifestyle and health-related variables, including smoking, alcohol consumption, diet, physical activity, disease history and family history of cancer, were self-reported and may be subject to recall, reporting and social desirability biases.

## Conclusion

The working population exhibits significant cancer risk factors, including high levels of unhealthy lifestyles and low levels of biological screening and vaccination. The variations in cancer risk factors across different populations and regions further emphasise the need for more targeted intervention strategies to address these concerns effectively.

## Supplementary material

10.1136/bmjopen-2025-107063 online supplemental file 1

10.1136/bmjopen-2025-107063 online supplemental file 2

## Data Availability

Data are available upon reasonable request.
