# Supplementary material for: Risk factors for cancer among the working population in China: a cross-sectional analysis of baseline data from the WECAN project
Source: BMJ Open. 2025 Dec 22;15(12):e107063. doi: 10.1136/bmjopen-2025-107063 (PMC12730758; doi:10.1136/bmjopen-2025-107063)
Supplement: online supplemental file 1 [file bmjopen-15-12-s001.docx]

**Baseline Assessment Tool for the Comprehensive Intervention Programme for Workplace Cancer Prevention in China**

# Part I Basic Information

1. **Gender:**

□ Male (questions in section 10 will be skipped).

□ Female

1. **Date of Birth:** |__|__|__|__| Year |__|__| Month |__|__| Date
2. **Ethnic group:**

□ Han Chinese

□ Other (go to question 1.3.1).

**1.3.1 Ethnicity:** Please specify _____________

1. **Education Level:**

□ Primary school education or below

□ Secondary school

□ Senior High School / Technical Secondary School / Technical School

□ Junior College Graduate

□ Bachelor's Degree Graduate

□ Postgraduate and Above

1. **Marital status:**

□ Single

□ Married or Cohabitation

□ Divorced or separated

□ Widowed

□ Other (go to question 1.5.1).

**1.5.1 Marital status:** Please specify ______

1. **Your current occupation:**

□ Director or senior of State Organs, Party and Mass Organizations, Enterprises, and Public Institutions

□ Clerical Staff and Related Personnel

□ Business and service industry personnel

□ Professional or technical personnel (such as doctor, lawyer, journalist, teacher, etc.).

□ Employee in agriculture, forestry, animal husbandry, fishery or water conservancy sectors

□ Equipment operators in manufacture and transportation sectors

□ Military personnel

□ Other (go to question 1.6.1).

**1.6.1 Your current occupation:** Please specify ______

# Part II Smoking Habits

1. **Do you have a smoking habit (having smoked continuously or cumulatively for 6 months or more, including cigarettes, tobacco leaves, and e-cigarettes)?**

□ Never (go to question 2.4).

□ Yes, and ongoing (go to question 2.1.2).

□ Used to, but have quit (go to question 2.1.1).

**2.1.1 How long did your most recent period of quitting smoking (completely stopping smoking altogether) last?**

|__|__|__|__| Year |__|__| Month (go to question 2.2).

**2.1.2 Which of the following description fits the best for your** **current thoughts** **on quitting smoking?**

□ Plan to quit smoking within a month

□ Consider quitting smoking within 6 months

□ Want to quit smoking, but not within 6 months

□ Don't want to quit smoking

□ I don't know

1. **After deducting the time of smoking cessation, how many years have you smoked in total? (Less than one year will be considered as one year).**

|__|__| year(s)

1. **How many cigarettes do you smoke on average per day (1 Liang tobacco leaves = 50 cigarettes; 1 e-cigarette cartridge ≈40 cigarettes [ Liang: Chinese unit of measurement, equivalent to 50 grams]) (If you have quit smoking, please fill in the information about your past smoking status)**

|__|__|__| . |__| cigarette(s)

1. **Under normal circumstances, are you exposed to second-hand smoke (inhaling smoke exhaled by smokers and smoke emitted from the end of cigarettes) every week?**

□ Yes (go to question 2.4.1)

□ Hardly ever (go to Part III Drinking Habits)

**2.4.1 If yes, how many days in a week that you are exposed to second-hand smoke for more than 15 minutes (within a single day):**

□ 0

□ 1

□ 2

□ 3

□ 4

□ 5

□ 6

□ 7

# Part III Drinking Habits

1. **Have you consumed any alcohol in the past 6 months?**

□ No (go to Part IV)

□ Yes, did more than 30 days ago

□ Yes, did in the past 30 days

1. **How often** **do you drink in the last 6 months?**

□ Every day

□ 5-6 days/week

□ 3-4 days/week

□ 1-2 days/week

□ 1-3 days/month

□ Less than 1 day/month

1. **Please recall whether you have consumed the following alcohol drinks on a regular basis in the past 6 months and estimate the frequency and amount consumed.**

|  |  | Drink or not?  0=No 1=Yes | Frequency of drinking (Choose one option only) | | | Typical amount of consumption per day (Liang) |
| --- | --- | --- | --- | --- | --- | --- |
|  |  |  | Days/week | Days/months | Days/half a year |  |
| 3.3.1 | Liquor (≥42 ABV) |  |  |  |  | □□□□  Liang |
| 3.3.2 | liquor (<42 ABV) |  |  |  |  | □□□□  Liang |
| 3.3.3 | Beer (500ml/bottle) |  |  |  |  | □□□□ bottle |
| 3.3.4 | Yellow rice wine |  |  |  |  | □□□□  Liang |
| 3.3.5 | White rice wine |  |  |  |  | □□□□  Liang |
| 3.3.6 | wine |  |  |  |  | □□□□  Liang |
| 3.3.7 | Barley wine |  |  |  |  | □□□□  Liang |
| 3.3.8 | Other, please specify ____________ |  |  |  |  | □□□□  Liang |

1. **Which of the following options fits the best your current thoughts about giving up drinking alcohol?**

□ Plan to give up drinking alcohol within a month

□ consider giving up drinking alcohol within 6 months

□ want to give up drinking alcohol, but not within 6 months

□ don't want to give up drinking alcohol

□ I don't know

# Part IV Physical Activity

**Note: The following questions ask about your participation in various types of physical activities (including those related to transportation, work, housework, and leisure) in one week over the past 6 months. Please answer based on your actual situation.**

**(A) Physical activity from transportation**

**4.1Do you go out by walking or cycling** **for at least 10 minutes?**

□ Yes (go to question 4.1.1).

□ No (go to question 4.2).

**4.1.1 How many days per week do you usually go out by walking or cycling for at least 10 minutes? (Days)**

□ 1

□ 2

□ 3

□ 4

□ 5

□ 6

□ 7

**4.1.2** **How long are you walking and/or cycling in a routine day?**

|__|__|__| minutes

*Note: Any single activity session lasting less than 10 minutes will not be counted.*

**(B) Physical activity at work (other than transportation**).

**4.2 Do you** **engage in** **moderate-intensity** **activities** **that** **last more than 10 minutes** **at** **work?**

**(Moderate-intensity activities are activities that cause a mild increase in breath and heartbeat, such as carrying lighter items**.**)**

□ Yes (go to question 4.2.1).

□ No (go to question 4.3).

**4.2.1 How many days per week do you usually engage in the above-mentioned moderate-intensity activities at work? (Days)**

□ 1

□ 2

□ 3

□ 4

□ 5

□ 6

□ 7

**4.2.2 How long on average do you usually engage in the above-mentioned medium-intensity activities in a single day?**

|__|__|__| Minutes

*Note: Any single activity session lasting less than 10 minutes will not be counted.*

**4.3 Do** **you** **engage in** **high-intensity** **activities** **that** **last more than 10 minutes** **at** **work?**

**(High-intensity activity refers to activities that require greater physical exertion, or cause a significant increase in breath and heartbeat, such as carrying heavy objects, etc.)**

□ Yes (go to question 4.3.1).

□ No (go to question 4.4).

**4.3.1 How many days of a week do you usually have the above-mentioned high-intensity activities at work? (Days)**

□ 1

□ 2

□ 3

□ 4

□ 5

□ 6

□ 7

**4.3.2 How long do you** **usually** **carry out the above-mentioned high-intensity activities per day at work?**

|__|__|__| Minutes

*Note: If the duration of activity is less than 10 minutes each time, it will not be counted.*

**(C) Housework-related physical activity (other than commuting and** **work).**

**4.4Do you engage in moderate-intensity housework activities in your daily life that lasts more than 10 minutes?**

**(Moderate-intensity activities are activities that cause a mild increase in breath and heartbeat, such as cleaning))**

□ Yes (go to question 4.4.1).

□ No (go to question 4.5).

**4.4.1 How many days do you usually engage in the above-mentioned moderate-intensity housework activities in a week?**

□ 1

□ 2

□ 3

□ 4

□ 5

□ 6

□ 7

**4.4.2 How long do you** **usually do the above-mentioned moderate-intensity housework activities in a day?**

|__|__|__| Minutes

*Note: If the duration of activity is less than 10 minutes each time, it will not be counted.*

**4.5Do you have high-intensity housework activities in your life that last more than 10 minutes?**

**(High-intensity activity is an activity that causes a significant increase in breath and heartbeat, such as carrying heavy objects)**

□ Yes (go to question 4.5.1).

□ No (go to question 4.6).

**4.5.1 How many days of a week do you usually do the above-mentioned high-intensity housework activities in your life?**

□ 1

□ 2

□ 3

□ 4

□ 5

□ 6

□ 7

**4.5.2 How much time do you usually spend on the above-mentioned high-intensity household chores in a day?**

|__|__|__| Minutes

*Note: If the duration of activity is less than 10 minutes each time, it will not be counted.*

**(D) Recreational physical activity (other than commuting,** **work, housework**).

**4.6Do** **you** **engage in** **moderate-intensity** **activities** **that last at least 10 minutes** **at leisure?**

**(Moderate-intensity activities are activities that cause a mild increase in breath and heartbeat,** **such as brisk walking, tai chi, etc.).**

□ Yes (go to question 4.6.1).

□ No (go to question 4.7).

**4.6.1 How many days of a week do you usually engage in the above-mentioned moderate-intensity activities when you are at leisure?**

□ 1

□ 2

□ 3

□ 4

□ 5

□ 6

□ 7

**4.6.2 How long do you usually do the above-mentioned moderate-intensity leisure time activities in a day?**

|__|__|__| Minutes (the number ranged from 10-72 0).

*Note: If the duration of activity is less than 10 minutes each time, it will not be counted.*

**4.7Do you have high-intensity activities that last at least 10 minutes at leisure?**

**(High-intensity activities refer to activities** that cause a significant increase in breath and heartbeat**, such as long-distance running, swimming, playing football, etc.).**

□ Yes (go to question 4.7.1).

□ No (go to question 4.8).

**4.7.1 How many** **days** **of** **a week do you** **usually** **engage in the above-mentioned high-intensity activities when you are at leisure?**

□ 1

□ 2

□ 3

□ 4

□ 5

□ 6

□ 7

**4.7.2 How long do you** **usually do the above-mentioned high-intensity leisure time activities in a day?**

|__|__|__| Minutes

*Note: If the duration of activity is less than 10 minutes each time, it will not be counted.*

**4.8 Which of the following options fits the best for your current physical activity status?**

□ Absolutely adequate

□ Adequate

□ Normal

□ Inadequate

□ Absolutely Inadequate

**4.9 Which of the following options fits the best for your thoughts on changing or adjusting your physical activity status?**

□ Plan to change or adjust physical activity status within a month

□ Consider changing or adjusting physical activity status within 6 months

□ Want to change or adjust physical activity status, but not within 6 months

□ Don't want to change or adjust physical activity status

□ I don't know

**(E) Total static behaviour**

**4.10 How long do you spend in a day sitting, leaning, or lying down? (Including time spent sitting at work, studying, reading, watching TV, using the computer, resting, and all other static behaviours, but not bedtime).**

|__|__| Hour(s) |__|__| minute(s)

**(F) Sleep behaviour**

**4.11 How long do you sleep in total in a day?**

|__|__| Hour(s) |__|__| minute(s)

**4.12 Which of the following options fits the best for your current weight?**

□ Significantly above normal

□ Above normal

□ Normal

□ Below normal

□ Significantly below normal

**4.13 Which of the following options best aligns with your current thoughts on weight management (including weight gain and weight loss)?**

□ Already taking measures

□ Planning to take measures to manage weight within one month

□ Considering taking measures to manage weight within six months

□ Want to take measures to manage weight, but not within six months

□ Do not want to take measures to manage weight

□ Not sure

# Part V Eating Habits

**5.1 What is your taste preference?**

□ Very salty

□ Slightly salty

□ Moderate

□ Slightly light taste

□ Very light taste

**5.2 What is the temperature of the food you prefer when eating** **or drinking water/beverages?**

□ Cold

□ Warm

□ Hot

**5.3 How many times in total have you consumed barbecued food in the past 6 months? (Barbecued food mainly refers to food cooked over an open flame, excluding food baked in a household oven or air fryer.)**

□ 0 times

□ 1-2 times

□ 3-5 times

□ 6-11 times

□ 12 times or more

**5.4 How frequently have you chewed betel nuts in the past 6 months?**

□ Every day

□ 5-6 days/week

□ 3-4 days/week

□ 1-2 days/week

□ 1-3 days/month

□ Less than 1 day/month

□ Never eat (go to question 5.5)

**5.4.1** **If so, how many pieces do you chew on average per day?** |__|__| pieces

**5.5 How often do you have breakfast in the past 6 months?**

□ Every day

□ 5-6 days/week

□ 3-4 days/week

□ 1-2 days/week

□ Less than 1 day/week

**5.6 How often do you eat late-night snacks in the past 6 months?**

□ Less than 1 day/week

□ 1-2 days/week

□ 3-4 days/week

□ 5-6 days/week

□ every day

**5.7 Over the past 6 months, how frequently do you dine out or order takeout?**

□ Less than 1 day/week

□ 1-2 days/week

□ 3-4 days/week

□ 5-6 days/week

□ Every day

**5.8 Please recall whether you have eaten the following foods in the past 6 months, and the frequency and amount of each type of food consumed (1 Liang is equivalent to 50 grams):**

|  |  | Ate or not:  0=No  1=Yes | Frequency of consumption (choose one of the options) | | | | Average amount of consumption each time |
| --- | --- | --- | --- | --- | --- | --- | --- |
|  |  |  | Times/Day | Times/Week | Times /month | Times/half a year |  |
| 5.8.1 | Mixed grains (millet/corn/oats/buckwheat/rye/sorghum/barley/barley.) /black rice/corn, etc.).  (raw/uncooked weight) |  |  |  |  |  | □□. □ Liang |
| 5.8.2 | Potatoes (sweet potatoes/potatoes/purple potatoes/taro/yam/cassava, etc.) (raw/uncooked weight). |  |  |  |  |  | □□. □ Liang |
| 5.8.3 | Miscellaneous beans (red beans/mung beans/black beans/kidney beans/flower beans/broad beans/peas/chickpeas, etc.).  (raw/uncooked weight) |  |  |  |  |  | □□. □ Liang |
| 5.8.4 | Fresh pork  (Recorded by weight) |  |  |  |  |  | □□. □ Liang |
| 5.8.5 | Fresh beef, lamb and other meat  (raw/uncooked weight) |  |  |  |  |  | □□. □ Liang |
| 5.8.6 | Processed meats (bacon/sausage/marinated meat/ham/bacon/marinated fish/pork floss, etc.). |  |  |  |  |  | □□. □ Liang |
| 5.8.7 | Fresh vegetables (including mushrooms) |  |  |  |  |  | □□. □ Liang |
| 5.8.8 | Fresh fruit |  |  |  |  |  | □□. □ Liang |
| 5.8.9 | Salted vegetables, pickled vegetables, sour vegetables, fermented bean curd, salted duck eggs, preserved eggs, etc. |  |  |  |  |  | □□. □ times |
| 5.8.10 | Salty snacks (potato chips, seaweed, sunflower seeds, spiced beans, etc.) |  |  |  |  |  | □□. □ times |

**5.9 Which of the following options fits the best for your current dietary pattern?**

□ Absolutely healthy

□ Healthy

□ Neutral

□ Unhealthy

□ Absolutely unhealthy

□ Not sure

**5.10 Which of the following options fits the best for your thoughts on changing or adjusting your eating habits?**

□ Plan to change or adjust eating habits within a month

□ Consider changing or adjusting eating habits within 6 months

□ Want to change or adjust eating habits, but not within 6 months

□ Don't have a plan to change or adjust eating habits

□ I don't know

**5.11 Which of the following options fits the best your current overall life situation?**

□ Very satisfied

□ Satisfied

□ Acceptable

□ dissatisfied

□ Very dissatisfied

# Part VI Exposure to Harmful Gases or Substances

**6.1 Who mainly does the cooking in your home?**

□ Myself

□ Spouse

□ Others

□ No cooking at home (go to question 6.6)

**6.2 Does your family use a range hood when cooking at home?**

□ Never

□ 1-2 days/week

□ 3-5 days/week

□ Almost every day

**6.3 Does your family open windows for ventilation when cooking at home, provided the weather permits?**

□ Never

□ Occasionally

□ Frequently

□ Always

**6.4 Are you in the habit of stir-frying over high heat or deep-frying at high temperatures when cooking? (Stir-frying over high heat mainly refers to heating the oil until it is very hot, even smoking.)**

□ Never

□ 1-3 days/month

□ 1-2 days/week

□ 3-5 days/week

□ Almost every day

**6.5 What is the situation of oil fumes in other rooms (excluding the kitchen) of your house when cooking?**

□ No oil fumes

□ Slight amount

□ Moderate amount

□ Heavy amount

**6.6 Have you been exposed to the following substances in your workplace for more than one year? (Multiple selections allowed)**

□ Asbestos

□ Beryllium, uranium, radon, chromium, cadmium, nickel, silicon, etc.

□ Diesel exhaust, coal smoke, coal dust

□ Others (Go to question 6.6.1)

□ No (go to Part VII)

**6.6.1 Substance exposure: Others,** please specify ____________

**6.7 What protective measures do you take when exposed to the above substances? (Multiple selections allowed)**

□ Face mask

□ Dust-proof or gas-proof mask/respirator

□ Protective clothing

□ Dust-proof helmet

□ Frequently change work clothes and take a bath

□ Others (Go to question 6.7.1)

□ No protection (go to Part VII)

**6.7.1 Protective measures:** Others, please specify ____________

# Part VII: History of Illness

**Do you have any of the following diseases (clearly diagnosed by a formal medical institution):**

**7.1 Have you been diagnosed with any of the following common chronic diseases? (Multiple selections allowed)**

□ Hypertension (Please complete 7.1.1)

□ Hyperlipidemia (Please complete 7.1.2)

□ Diabetes (Please complete 7.1.3)

□ Coronary heart disease

□ Heart failure

□ Stroke

□ Others (Go to question 7.1.4)

□ None of the above (Go to question 7.2)

**7.1.1 In the past two weeks, have you taken any antihypertensive drugs?**

□ Yes

□ No

**7.1.2 Are you currently taking any lipid-lowering drugs?**

□ Yes

□ No

**7.1.3 Are you currently taking any hypoglycaemic drugs or using insulin?**

□ Yes

□ No

**7.1.4 Common chronic diseases: Others, please specify: ____________**

**7.2 Have you been diagnosed with any of the following chronic respiratory diseases? (Multiple selections allowed)**

□ Chronic bronchitis

□ Emphysema

□ Asthma

□ Silicosis or pneumoconiosis

□ Tuberculosis

□ Others (go to question 7.2.1)

□ None of the above (go to question 7.3)

**7.2.1 Chronic respiratory diseases:** Others, please specify: ____________

**7.3 Have you been diagnosed with any of the following upper gastrointestinal diseases? (Multiple selections allowed)**

□ Esophageal intraepithelial neoplasia

□ Chronic atrophic gastritis

□ Hypertrophic gastritis

□ Gastric polyps

□ Postoperative residual stomach

□ Gastric mucosal intraepithelial neoplasia

□ Gastrointestinal metaplasia

□ Others (go to question 7.3.1)

□ None of the above (go to question 7.4)

**7.3.1 Upper gastrointestinal diseases:** Others, please specify: ____________

**7.4 Have you been diagnosed with any of the following liver diseases? (Multiple selections allowed)**

□ Chronic hepatitis B

□ Chronic hepatitis C

□ Liver cirrhosis

□ Others (go to question 7.4.1)

□ None of the above (go to question 7.5)

**7.4.1 Liver diseases:** Others, please specify: ____________

**7.5 Have you been diagnosed with any of the following intestinal diseases? (Multiple selections allowed)**

□ Colorectal polyps

□ Lynch syndrome

□ Inflammatory bowel disease (Crohn's disease, ulcerative colitis, etc.)

□ Others (go to question 7.5.1)

□ None of the above (go to Part Ⅷ)

**7.5.1 Intestinal diseases:** Others, please specify: ____________

# Part Ⅷ: Family Medical History

**8.1 Has any of your first-degree relatives (including parents, full siblings, children) ever been diagnosed with cancer?**

□ Yes

□ No

**8.1.1 If yes, please complete the form below:**

|  | **0= No; 1=Yes** | **Number of confirmed cases** | **Age at earliest diagnosis** |
| --- | --- | --- | --- |
| lung cancer |  |  |  |
| oesophageal cancer |  |  |  |
| gastric cancer |  |  |  |
| hepatocarcinoma |  |  |  |
| Cancer of the colon (rectum) |  |  |  |
| Pancreatic cancer |  |  |  |
| Prostate cancer |  |  |  |
| Thyroid cancer |  |  |  |
| Breast cancer |  |  |  |
| Cervical cancer |  |  |  |
| Ovarian cancer |  |  |  |
| Other (including unclear) |  |  |  |

**8.2 Has any of your second-degree relatives (including grandparents, uncles, aunts, and great-uncles/great-aunts) ever been diagnosed with cancer?**

□ Yes

□ No

**8.2.1 If yes, please complete the form below:**

|  | **0= No; 1=Yes** | **Number of confirmed cases** | **Age at earliest diagnosis** |
| --- | --- | --- | --- |
| lung cancer |  |  |  |
| oesophageal cancer |  |  |  |
| gastric cancer |  |  |  |
| hepatocarcinoma |  |  |  |
| Cancer of the colon (rectum) |  |  |  |
| Pancreatic cancer |  |  |  |
| Prostate cancer |  |  |  |
| Thyroid cancer |  |  |  |
| Breast cancer |  |  |  |
| Cervical cancer |  |  |  |
| Ovarian cancer |  |  |  |
| Other (including unclear) |  |  |  |

# Part Ⅸ Biological Testing

**9.1Have you ever tested for hepatitis B surface antigen (HBsAg)?**

□ No (go to question 9.2)

□ Yes (go to question 9.1.1)

**9.1.1 If yes, what was the result?**

□ Negative

□ Positive

□ Unclear

**9.1.2** **The time of the most recently test: |__|__|__|__| year**

**9.2 Have you ever been vaccinated against hepatitis B?**

□ Yes

□ No

**9.3 Have you been tested for Helicobacter pylori** **(HP)?**

□ No (go to question 9.4)

□ Yes (go to question 9.3.1)

**9.3.1 If yes, what was the result?**

□ Negative

□ Positive

□ Unclear

**9.3.2** **The time of the most recently test: |__|__|__|__| year**

**9.4 Have you ever had a faecal occult blood test (FOBT)?**

□ No (go to question 9.5)

□ Yes (go to question 9.4.1)

**9.4.1 If yes, what was the result?**

□ Negative

□ Positive

□ Unclear

**9.4.2** **The time of the most recently test: |__|__|__|__| year**

**9.5 Have you ever had a fasting venous blood glucose test (FBG)?**

□ Yes

□ No (go to question 9.6)

**9.5.1 If yes, please provide your last measurement of fasting venous blood sugar (FBG).**

Date: |__|__|__|__|Year |__|__|Month |__|__|day

Result: |__|__|. |__|__|mmol/L

**Source of result:**

□ Physical examination/Hospital check-up

□ Self-test

**9.6 Have you ever had a glycated haemoglobin test?**

□ Yes

□ No (go to question 9.7)

**9.6.1 If yes, please recall your most recent glycated haemoglobin (HbA1c)**

Date: |__|__|__|__|Year |__|__|Month |__|__| day

Result: |__|__|. |__|%

**9.7 Have you ever had a blood lipid test?**

□ Yes

□ No (go to Part X)

**9.7.1 If yes, Please provide your last measurement of** **blood lipids**

Date: |__|__|__|__|Year |__|__|Month |__|__|day

Results: Total cholesterol (TC): |__|__|. |__|__|mmol/L

Low-density lipoprotein (LDL): |__|__|. |__|__|mmol/L

High-density lipoprotein (HDL): |__|__|. |__|__|mmol/L

Triglycerides (TG): |__|__|. |__|__|mmol/L

**(Male respondents skip to Part XI)**

# Part X Physiological Aspects of Female Fertility

**10.1 The age of your menarche:** |__|__| years old

**10.2 Is your menstrual cycle regular?**

□ No

□ Yes

**10.3 Are you menopausal/amenorrhea?**

□ No (Go to question 10 4）

□ Yes (go to question 10.3.1).

**10.3.1** **If so, your age of menopause/amenorrhea is:** |__|__| years old

**10.4 Have you ever given birth (both naturally and by caesarean section)?**

□ No (Go to question 10.5）

□ Yes (go to question 10.4.1).

**10.4.1 If yes, your initial childbearing age is:** |__|__| years old

**10.5 Do you have a history of breastfeeding?**

□ No (Go to question 10.6）

□ Yes (go to question 10.5.1).

**10.5.1** **If so, how long in total did you breastfeed for (less than** **one month is counted as one month)?**  |__|__| month(s)

**10.6 Have you ever had a benign breast disease (breast hyperplasia, nodules, ductal dilation, benign fibroadenoma, infection, etc.)?**

□ No (Go to question 10.7）

□ Yes (go to question 10.6.1).

**10.6.1** **If** **so, please indicate the name of the disease:** ________________

**10.7 Have you ever had surgery on the female reproductive system** **(surgery for parts of the uterus, ovaries, and fallopian tubes, including caesarean section)?**

□ No (Go to question 10.8）

□ Yes (go to question 10.7.1).

**10.7.1** **If** **yes, please specify the name of the surgery or place on the body of surgery:** ______________

**10.8 Have you been vaccinated against human papillomavirus** **(HPV)?**

□ No

□ Yes

# Part XI Anthropometric measurements

**11.1 Height:** |___|____|____| Centimetres (cm)

Quality Control Photo (Side View of Subject's Height Measurement)

**11.2 Weight:** |__|__|__|. |__|Kilograms (kg)

**11.3 Waist:** |__|__|__|. |__| Centimetres (cm)

**11.4 Special Circumstances Description:** _________________ (Not Mandatory)

# Part XII Blood Pressure Measurement

**12.1 Temperature of the Physical Examination Site:**

**12.1.1 Indoor Temperature: ______**

**12.1.2 Outdoor Temperature: ______**

**12.2 Measurement of Blood Pressure and Heart Rate**

**12.2.1 First Systolic Blood Pressure (mmHg): |__|__|__|**

**12.2.2 First Diastolic Blood Pressure (mmHg): |__|__|__|**

**12.2.3 First Heart Rate (beats/min):|__|__|__|**

**12.2.4 Quality Control Photo (Side View of Subject in Sitting Posture)**

**12.2.5 Second Systolic Blood Pressure (mmHg): |__|__|__|**

**12.2.6 Second Diastolic Blood Pressure (mmHg): |__|__|__|**

**12.2.7 Second Heart Rate (beats/min):|__|__|__|**

**12.2.8 Quality Control Photo (Photo of Sphygmomanometer Screen with Stable Blood Pressure Reading)**

**12.2.9 Third Systolic Blood Pressure (mmHg): |__|__|__|**

**12.2.10 Third Diastolic Blood Pressure (mmHg): |__|__|__|**

**12.2.11 Third Heart Rate (beats/min): |__|__|__|**

**12.2.12 Quality Control Photo (Photo of Sphygmomanometer Screen with Stable Blood Pressure Reading)**

**12.3 Special Circumstances Description: _________________ (Not Mandatory)**
