# Supplementary material for: Risk factors for cancer among the working population in China: a cross-sectional analysis of baseline data from the WECAN project
Source: BMJ Open. 2025 Dec 22;15(12):e107063. doi: 10.1136/bmjopen-2025-107063 (PMC12730758; doi:10.1136/bmjopen-2025-107063)
Supplement: online supplemental file 2 [file bmjopen-15-12-s002.docx]

**Table S1 Associations between participant characteristics and dietary cancer risk factors: multivariable-adjusted odds ratios [OR (95% CI)]^a^**

| **Characteristics** | **Dietary cancer risk factors** | | | | | |
| --- | --- | --- | --- | --- | --- | --- |
|  | **Insufficient vegetable intake** | **Insufficient fruit intake** | **Low dietary fiber intake** | **High-salt diet** | **Excessive red meat consumption** | **Frequent consumption of processed meat** |
| **Sex** (male vs. female) | 1.2(0.9-1.6) | 3.1(2.2-4.4) | 1.4(1.0-1.9) | 2.5(1.2-5.1) | 1.7(1.2-2.3) | 1.6(1.1-2.4) |
| **Work type** (blue-collar vs. white-collar) | 1.0(0.7-1.4) | 1.2(0.8-1.7) | 1.1(0.8-1.5) | 0.7(0.3-1.5) | 1.0(0.7-1.3) | 1.2(0.8-1.8) |
| **Age group** (>=40 vs. <40) | 0.9(0.6-1.2) | 0.9(0.6-1.3) | 0.9(0.6-1.2) | 0.4(0.2-0.8) | 0.7(0.5-1.0) | 0.8(0.5-1.2) |
| **Education** (vs. junior high school or below) |  |  |  |  |  |  |
| high school ^b^ | 1.0(0.6-1.7) | 1.0(0.5-1.9) | 1.2(0.7-2.0) | 1.5(0.5-4.9) | 1.0(0.6-1.6) | 1.1(0.5-2.2) |
| junior college | 1.3(0.8-2.1) | 0.7(0.4-1.3) | 1.4(0.8-2.3) | 0.7(0.3-2.0) | 0.9(0.6-1.5) | 1.4(0.7-2.7) |
| bachelor's degree or above | 1.1(0.6-1.9) | 0.7(0.4-1.4) | 1.2(0.7-2.1) | 1.5(0.4-5.1) | 0.8(0.5-1.5) | 1.1(0.6-2.3) |
| **Marital status** (unmarried/divorced/widowed vs. married/cohabiting) | 1.1(0.8-1.7) | 1.3(0.8-2.1) | 1.4(0.9-2.1) | 2.3(0.5-10.4) | 1.0(0.6-1.5) | 1.4(0.9-2.1) |
| **Family history of cancer** (yes vs. no) | 1.1(0.8-1.5) | 1.3(0.9-1.9) | 1.2(0.9-1.6) | 0.8(0.4-1.7) | 1.0(0.7-1.4) | 1.1(0.8-1.6) |
| **City** (vs. Nanchong) |  |  |  |  |  |  |
| Wuhai | 1.5(1.0-2.2) | 2.0(1.2-3.2) | 1.8(1.2-2.7) | 0.8(0.3-2.1) | 0.6(0.4-1.0) | 0.7(0.4-1.1) |
| Xiangtan | 1.0(0.7-1.4) | 2.5(1.7-3.8) | 1.2(0.8-1.7) | 0.8(0.3-1.9) | 1.1(0.8-1.7) | 1.7(1.1-2.5) |

^a^ Multivariable logistic regression model was performed for each cancer risk factor, with covariates including sex, work type, age group, education, marital status, family history of cancer, and city. All covariates were simultaneously adjusted for each other.

^b^ High school included vocational school and technical school.

**Table S2 Comparison of cancer risk factors among different populations**

| **Characteristics** | | **Smoking** | | **Alcohol consumption** | | **Physical inactivity** | |
| --- | --- | --- | --- | --- | --- | --- | --- |
|  |  | **Number (Rate)** | **χ^2^ value**  **(*P* value)** | **Number (Rate)** | **χ^2^ value**  **(*P* value)** | **Number (Rate)** | **χ^2^ value**  **(*P* value)** |
| **Sex** | male | 298(57.8) | 250.39(*P*<0.001) | 386(74.8) | 72.25(*P*<0.001) | 248(48.1) | 0.58(*P*=0.445) |
|  | female | 12(3.7) |  | 149(45.9) |  | 165(50.8) |  |
| **Work type** | white-collar | 95(26.5) | 28.55(*P*<0.001) | 215(60.1) | 3.41(*P*=0.065) | 173(48.3) | 0.15(*P*=0.695) |
|  | blue-collar | 215(44.5) |  | 320(66.3) |  | 240(49.7) |  |
| **Age group (years)** | <40 | 123(30.9) | 11.52(*P*<0.001) | 261(65.6) | 1.26(*P*=0.262) | 200(50.3) | 0.40(P=0.530) |
|  | >=40 | 187(42.2) |  | 274(61.9) |  | 213(48.1) |  |
| **Education** | junior high school or below | 54(48.7) | 32.47(*P*<0.001) | 46(41.4) | 31.89(*P*<0.001) | 61(55.0) | 1.81(*P*=0.612) |
|  | high school / vocational school / technical school | 74(46.3) |  | 96(60.0) |  | 76(47.5) |  |
|  | junior college | 105(40.1) |  | 177(67.6) |  | 126(48.1) |  |
|  | bachelor's degree or above | 77(25.0) |  | 216(70.1) |  | 150(48.7) |  |
| **Marital status** | married/cohabiting | 260(37.9) | 1.73(*P*=0.188) | 431(62.8) | 1.00(*P*=0.318) | 332(48.4) | 0.75(*P*=0.385) |
|  | unmarried/divorced/widowed | 50(32.3) |  | 104(67.1) |  | 81(52.3) |  |
| **Family history of cancer** | no | 199(37.2) | 0.07(*P*=0.790) | 333(62.2) | 1.20(*P*=0.274) | 257(48.0) | 0.67(*P*=0.411) |
|  | yes | 111(36.3) |  | 202(66.0) |  | 156(51.0) |  |
| **City** | Wuhai | 105(37.5) | 4.00(*P*=0.136) | 129(46.1) | 68.93(*P*<0.001) | 156(55.7) | 14.88(*P*<0.001) |
|  | Nanchong | 114(40.6) |  | 224(79.7) |  | 145(51.6) |  |
|  | Xiangtan | 91(32.5) |  | 182(65.0) |  | 112(40.0) |  |

**Table S2 Comparison of cancer risk factors among different populations (Continued)**

| **Characteristics** | | **Obesity** | | **Central obesity** | | **Insufficient vegetable intake** | | **Insufficient fruit intake** | |
| --- | --- | --- | --- | --- | --- | --- | --- | --- | --- |
|  |  | **Number (Rate)** | **χ^2^ value**  **(*P* value)** | **Number (Rate)** | **χ^2^ value**  **(*P* value)** | **Number (Rate)** | **χ^2^ value**  **(*P* value)** | **Number (Rate)** | **χ^2^ value**  **(*P* value)** |
| **Sex** | male | 146(28.3) | 14.18(*P*<0.001) | 237(45.9) | 48.50(*P*<0.001) | 314(60.9) | 1.28(*P*=0.259) | 431(83.5) | 48.93(*P*<0.001) |
|  | female | 55(16.9) |  | 72(22.2) |  | 185(56.9) |  | 202(62.2) |  |
| **Work type** | white-collar | 84(23.5) | 0.07(*P*=0.798) | 115(32.1) | 5.72(*P*=0.017) | 209(58.4) | 0.24(*P*=0.628) | 250(69.8) | 9.89(*P*=0.002) |
|  | blue-collar | 117(24.2) |  | 194(40.2) |  | 290(60.0) |  | 383(79.3) |  |
| **Age group (years)** | <40 | 88(22.1) | 1.33(*P*=0.249) | 127(31.9) | 7.59(*P*=0.006) | 244(61.3) | 1.22(*P*=0.270) | 303(76.1) | 0.30(*P*=0.582) |
|  | >=40 | 113(25.5) |  | 182(41.1) |  | 255(57.6) |  | 330(74.5) |  |
| **Education** | junior high school or below | 41(36.9) | 19.45(*P*<0.001) | 62(55.9) | 33.00(*P*<0.001) | 66(59.5) | 1.82(*P*=0.610) | 89(80.2) | 5.95(*P*=0.114) |
|  | high school / vocational school / technical school | 46(28.8) |  | 71(44.4) |  | 92(57.5) |  | 129(80.6) |  |
|  | junior college | 60(22.9) |  | 91(34.7) |  | 164(62.6) |  | 193(73.7) |  |
|  | bachelor's degree or above | 54(17.5) |  | 85(27.6) |  | 177(57.5) |  | 222(72.1) |  |
| **Marital status** | married/cohabiting | 162(23.6) | 0.17(*P*=0.684) | 256(37.3) | 0.53(*P*=0.466) | 402(58.6) | 0.83(*P*=0.362) | 507(73.9) | 3.70(*P*=0.054) |
|  | unmarried/divorced/widowed | 39(25.2) |  | 53(34.2) |  | 97(62.6) |  | 126(81.3) |  |
| **Family history of cancer** | no | 141(26.4) | 4.87(*P*=0.027) | 203(37.9) | 0.91(*P*=0.339) | 319(59.6) | 0.05(*P*=0.820) | 404(75.5) | 0.05(*P*=0.827) |
|  | yes | 60(19.6) |  | 106(34.6) |  | 180(58.8) |  | 229(74.8) |  |
| **City** | Wuhai | 105(37.5) | 44.14(*P*<0.001) | 140(50.0) | 37.46(*P*<0.001) | 181(64.6) | 4.91(*P*=0.086) | 220(78.6) | 22.49(*P*<0.001) |
|  | Nanchong | 42(15.0) |  | 71(25.3) |  | 159(56.6) |  | 184(65.5) |  |
|  | Xiangtan | 54(19.3) |  | 98(35.0) |  | 159(56.8) |  | 229(81.8) |  |

**Table S2 Comparison of cancer risk factors among different populations (Continued)**

| **Characteristics** | | **Low dietary fiber intake** | | **High-salt diet** | | **Excessive red meat consumption** | | **Frequent consumption of processed meat** | |
| --- | --- | --- | --- | --- | --- | --- | --- | --- | --- |
|  |  | **Number (Rate)** | **χ^2^ value**  **(*P* value)** | **Number (Rate)** | **χ^2^ value**  **(*P* value)** | **Number (Rate)** | **χ^2^ value**  **(*P* value)** | **Number (Rate)** | **χ^2^ value**  **(*P* value)** |
| **Sex** | male | 319(61.8) | 6.06(*P*=0.014) | 500(96.9) | 5.35(*P*=0.021) | 356(69.0) | 10.84(*P*=0.001) | 133(25.8) | 8.36(*P*=0.004) |
|  | female | 173(53.2) |  | 304(93.5) |  | 188(57.9) |  | 56(17.2) |  |
| **Work type** | white-collar | 198(55.3) | 2.62(*P*=0.106) | 343(95.8) | 0.07(*P*=0.799) | 226(63.1) | 0.66(*P*=0.416) | 70(19.6) | 3.05(*P*=0.081) |
|  | blue-collar | 294(60.9) |  | 461(95.5) |  | 318(65.8) |  | 119(24.6) |  |
| **Age group (years)** | <40 | 244(61.3) | 2.45(*P*=0.118) | 389(97.7) | 8.21(*P*=0.004) | 271(68.1) | 3.84(*P*=0.050) | 105(26.4) | 6.63(*P*=0.010) |
|  | >=40 | 248(56.0) |  | 415(93.7) |  | 273(61.6) |  | 84(19.0) |  |
| **Education** | junior high school or below | 64(57.7) | 2.13(*P*=0.546) | 104(93.7) | 5.31(*P*=0.150) | 66(59.5) | 1.70(*P*=0.638) | 16(14.4) | 5.99(*P*=0.112) |
|  | high school / vocational school / technical school | 94(58.8) |  | 154(96.3) |  | 103(64.4) |  | 33(20.6) |  |
|  | junior college | 162(61.8) |  | 246(93.9) |  | 171(65.3) |  | 66(25.2) |  |
|  | bachelor's degree or above | 172(55.8) |  | 300(97.4) |  | 204(66.2) |  | 74(24.0) |  |
| **Marital status** | married/cohabiting | 389(56.7) | 4.95(*P*=0.026) | 651(94.9) | 4.37(*P*=0.037) | 439(64.0) | 0.78(*P*=0.378) | 141(20.6) | 7.87(*P*=0.005) |
|  | unmarried/divorced/widowed | 103(66.5) |  | 153(98.7) |  | 105(67.7) |  | 48(31.0) |  |
| **Family history of cancer** | no | 316(59.1) | 0.19(*P*=0.661) | 513(95.9) | 0.29(*P*=0.591) | 342(63.9) | 0.37(*P*=0.542) | 116(21.7) | 0.53(*P*=0.467) |
|  | yes | 176(57.5) |  | 291(95.1) |  | 202(66.0) |  | 73(23.9) |  |
| **City** | Wuhai | 182(65.0) | 8.86(*P*=0.012) | 266(95.0) | 0.41(*P*=0.816) | 161(57.5) | 10.55(*P*=0.005) | 41(14.6) | 25.42(*P*<0.001) |
|  | Nanchong | 148(52.7) |  | 270(96.1) |  | 186(66.2) |  | 58(20.6) |  |
|  | Xiangtan | 162(57.9) |  | 268(95.7) |  | 197(70.4) |  | 90(32.1) |  |

**Table S2 Comparison of cancer risk factors among different populations (Continued)**

| **Characteristics** | | **Preference for hot foods** | | **Betel quid chewing** | | **Cooking-related household air pollution exposure** | | **Unprotected occupational exposure (>1 year)** | |
| --- | --- | --- | --- | --- | --- | --- | --- | --- | --- |
|  |  | **Number (Rate)** | **χ^2^ value**  **(*P* value)** | **Number (Rate)** | **χ^2^ value**  **(*P* value)** | **Number (Rate)** | **χ^2^ value**  **(*P* value)** | **Number (Rate)** | **χ^2^ value**  **(*P* value)** |
| **Sex** | male | 25(4.8) | 0.06(*P*=0.803) | 117(22.7) | 13.22(*P*<0.001) | 39(7.6) | 0.60(*P*=0.438) | 42(8.1) | 10.10(*P*=0.002) |
|  | female | 17(5.2) |  | 41(12.6) |  | 20(6.2) |  | 9(2.8) |  |
| **Work type** | white-collar | 15(4.2) | 0.85(*P*=0.357) | 56(15.6) | 4.04(*P*=0.044) | 21(5.9) | 1.26(*P*=0.261) | 11(3.1) | 9.79(*P*=0.002) |
|  | blue-collar | 27(5.6) |  | 102(21.1) |  | 38(7.9) |  | 40(8.3) |  |
| **Age group (years)** | <40 | 10(2.5) | 9.81(*P*=0.002) | 96(24.1) | 14.09(*P*<0.001) | 26(6.5) | 0.27(*P*=0.603) | 26(6.5) | 0.29(*P*=0.590) |
|  | >=40 | 32(7.2) |  | 62(14.0) |  | 33(7.5) |  | 25(5.6) |  |
| **Education** | junior high school or below | 12(10.8) | 11.78(*P*=0.008) | 6(5.4) | 19.25(*P*<0.001) | 18(16.2) | 16.60(*P*=0.001) | 2(1.8) | 9.95(*P*=0.019) |
|  | high school / vocational school / technical school | 9(5.6) |  | 41(25.6) |  | 9(5.6) |  | 17(10.6) |  |
|  | junior college | 13(5.0) |  | 56(21.4) |  | 15(5.7) |  | 13(5.0) |  |
|  | bachelor's degree or above | 8(2.6) |  | 55(17.9) |  | 17(5.5) |  | 19(6.2) |  |
| **Marital status** | married/cohabiting | 38(5.5) | 2.33(*P*=0.127) | 116(16.9) | 8.6(*P*=0.003) | 46(6.7) | 0.55(*P*=0.459) | 44(6.4) | 0.80(*P*=0.371) |
|  | unmarried/divorced/widowed | 4(2.6) |  | 42(27.1) |  | 13(8.4) |  | 7(4.5) |  |
| **Family history of cancer** | no | 31(5.8) | 1.99(*P*=0.159) | 100(18.7) | 0.01(*P*=0.925) | 37(6.9) | 0.02(*P*=0.881) | 23(4.3) | 8.04(*P*=0.005) |
|  | yes | 11(3.6) |  | 58(19.0) |  | 22(7.2) |  | 28(9.2) |  |
| **City** | Wuhai | 25(8.9) | 15.52(*P*<0.001) | 1(0.4) | 360.82(*P*<0.001) | 30(10.7) | 12.08(*P*=0.002) | 1(0.4) | 24.11(*P*<0.001) |
|  | Nanchong | 12(4.3) |  | 3(1.1) |  | 20(7.1) |  | 26(9.3) |  |
|  | Xiangtan | 5(1.8) |  | 154(55.0) |  | 9(3.2) |  | 24(8.6) |  |

**Table S2 Comparison of cancer risk factors among different populations (Continued)**

| **Characteristics** | | **Never-tested for HBsAg** | | **Never-tested for HP** | | **Unvaccinated against HBV** | | **Unvaccinated against HPV among females ≤ 45 years** | |
| --- | --- | --- | --- | --- | --- | --- | --- | --- | --- |
|  |  | **Number (Rate)** | **χ^2^ value**  **(*P* value)** | **Number (Rate)** | **χ^2^ value**  **(*P* value)** | **Number (Rate)** | **χ^2^ value**  **(*P* value)** | **Number (Rate)** | **χ^2^ value**  **(*P* value)** |
| **Sex** | male | 260(50.4) | 8.65(*P*=0.003) | 299(58.0) | 4.89(*P*=0.027) | 159(30.8) | 5.01(*P*=0.025) |  | - |
|  | female | 130(40.0) |  | 163(50.2) |  | 77(23.7) |  | - |  |
| **Work type** | white-collar | 138(38.6) | 15.35(*P*<0.001) | 184(51.4) | 3.15(*P*=0.076) | 80(22.4) | 10.09(*P*=0.002) | 82(59.0) | 3.05(*P*=0.081) |
|  | blue-collar | 252(52.2) |  | 278(57.6) |  | 156(32.3) |  | 66(70.2) |  |
| **Age group (years)** | <40 | 177(44.5) | 1.10(*P*=0.295) | 237(59.6) | 6.50(*P*=0.011) | 85(21.4) | 16.83(*P*<0.001) | 100(59.9) | 3.37(*P*=0.066) |
|  | >=40 | 213(48.1) |  | 225(50.8) |  | 151(34.1) |  | 48(72.7) |  |
| **Education** | junior high school or below | 83(74.8) | 58.94(*P*<0.001) | 90(81.1) | 48.96(*P*<0.001) | 52(46.9) | 39.79(*P*<0.001) | 9(56.3) | 8.02(*P*=0.046) |
|  | high school / vocational school / technical school | 88(55.0) |  | 99(61.9) |  | 53(33.1) |  | 19(67.9) |  |
|  | junior college | 112(42.8) |  | 137(52.3) |  | 78(29.8) |  | 61(74.4) |  |
|  | bachelor's degree or above | 107(34.7) |  | 136(44.2) |  | 53(17.2) |  | 59(55.1) |  |
| **Marital status** | married/cohabiting | 310(45.2) | 2.10(*P*=0.148) | 356(51.9) | 13.89(*P*<0.001) | 204(29.7) | 5.18(*P*=0.023) | 128(67.4) | 6.58(*P*=0.010) |
|  | unmarried/divorced/widowed | 80(51.6) |  | 106(68.4) |  | 32(20.7) |  | 20(46.5) |  |
| **Family history of cancer** | no | 266(49.7) | 6.62(*P*=0.010) | 323(60.4) | 17.57(*P*<0.001) | 160(29.9) | 2.48(*P*=0.115) | 94(69.1) | 4.42(*P*=0.036) |
|  | yes | 124(40.5) |  | 139(45.4) |  | 76(24.8) |  | 54(55.7) |  |
| **City** | Wuhai | 175(62.5) | 50.09(*P*<0.001) | 211(75.4) | 214.78(*P*<0.001) | 121(43.2) | 49.67(*P*<0.001) | 53(75.7) | 6.56(*P*=0.038) |
|  | Nanchong | 93(33.1) |  | 55(19.6) |  | 65(23.1) |  | 42(56.8) |  |
|  | Xiangtan | 122(43.6) |  | 196(70.0) |  | 50(17.9) |  | 53(59.6) |  |

**Table S3 Age- and sex-standardized ^a^ cancer risk factor rates**

| **Cancer Risk Factor** | **Standardized Rate (%)** |
| --- | --- |
| **Smoking** | 35.5 |
| **Alcohol consumption** | 60.9 |
| **Physical inactivity ^b^** | 48.6 |
| **Overweight/Obesity ^c^** | 60.2 |
| **Central obesity ^d^** | 36.2 |
| **Unhealthy diet ^e^** | 96.0 |
| insufficient vegetable intake | 60.2 |
| insufficient fruit intake | 74.7 |
| low dietary fiber intake | 59.6 |
| high-salt diet | 95.8 |
| excessive red meat consumption | 64.7 |
| frequent consumption of processed meat | 21.7 |
| **Preference for hot foods ^f^** | 5.4 |
| **Betel quid chewing** | 18.5 |
| **Cooking-related household air pollution exposure ^g^** | 7.8 |
| **Unprotected occupational exposure (>1 year)** | 5.9 |
| **HBsAg testing Rate** | 51.9 |
| **HP testing Rate** | 42.2 |
| **Unvaccinated against HBV** | 28.3 |

Abbreviations: HBsAg, hepatitis B surface antigen; HP, Helicobacter pylori; HBV, hepatitis B virus.

Notes:

^a^ Standardization was performed using the age- and sex-specific distribution of the employed population from China's 7th National Population Census as the standard population.

^b^ Physical inactivity: <150 minutes of moderate-intensity or <75 minutes of high-intensity physical activity per week.

^c^ Overweight/obesity: BMI (body mass index) ≥ 24.0 kg/m².

^d^ Central obesity: waist circumference ≥90 cm for men and ≥85 cm for women.

^e^ Unhealthy diet: meeting ≥2 of the following: insufficient vegetable intake (< 300 g/day), insufficient fruit intake (< 200 g/day), low dietary fiber intake (< 25 g/day), excessive red meat consumption (> 500 g/week), high-salt diet (moderate to salty taste preferences, or the consumption of takeout meals, processed meats, pickled vegetables, fermented bean curd, or salty snacks at least once per week), and frequent consumption of processed meat (consumption of processed meat at least once per week).

^f^ Preference for hot foods: frequent consumption of foods served at high temperatures.

^g^ Cooking-related household air pollution exposure: cooking with high heat (e.g., stir-frying or deep-frying) without frequent use of a range hood.

**Table S4 Comparison of lifestyle-related cancer risk factor prevalences between the current study and prior studies in China**

| **Risk Factor** | **Definition / Indicator** | **Current Study** | **Previous Studies on working populations** | **2018 National Survey on general population ^d^** |
| --- | --- | --- | --- | --- |
| **Smoking** | Current smoking | 35.5% | 24.7% ^a^ | 26.2% |
| **Alcohol consumption** | **Current study:** drinking alcohol within the past 6 months  **Previous study and National survey:** drinking alcohol within the past 12 months | 60.9% | 39.3% ^a^ | 39.8% |
| **Physical inactivity** | Engaging in <150 min of moderate-intensity or <75 min of vigorous-intensity physical activity per week | 48.6% | 32.0% ^b^ | 22.3% |
| **Overweight/**  **Obesity** | BMI (body mass index) ≥ 24.0 kg/m² | 60.2% | 42.3% ^c^ | 50.7% |
| **Central obesity** | **Current study / National survey:** waist circumference ≥90 cm for men and ≥85 cm for women. **Previous study:** waist circumference ≥85 cm for men and ≥80 cm for women. | 36.2% | 50.6% ^c^ | 35.2% |

Notes:

a. The 2009 study was a large-scale investigation that included 13,492 participants aged 18–60 years (mean age 38.7 ± 10.3 years, 51.1% male), consisting of civil servants, scientific and technical personnel, administrative staff, teachers, and physical workers from six provinces across eastern, central, and western China. (Zhonghua Yu Fang Yi Xue Za Zhi / Chinese Journal of Preventive Medicine, 2012, 53(2): 212-217)

b. The 2021 study surveyed 11,748 on-the-job employees of China Railway Chengdu Bureau Group Co., Ltd. (mean age 37.8 ± 10.7 years, 89.1% male) from three southwestern provinces of China—Sichuan, Guizhou, and Chongqing. (DOI: 10.3760/cma.j.cn112338-20230715-00010)

c. The 2016 study was based on 12,368 employees aged 18–60 years (mean age 41.2 ± 9.0 years, 41.7% male) from national comprehensive demonstration zones for chronic disease prevention and control across China. (Zhonghua Yu Fang Yi Xue Za Zhi / Chinese Journal of Preventive Medicine, 2019, 46(3): 214-219)

d. Data were obtained from the 2018 China Chronic Disease and Risk Factor Surveillance, a national survey covering 302 monitoring sites and including 184,509 permanent residents aged ≥ 18 years (mean age 43.8 years, 44.4% male). (Report on Chronic Disease Risk Factor Surveillance in China, 2018)
