# Supplementary material for: Risk factors for cancer among the working population in China: a cross-sectional analysis of baseline data from the WECAN project
Source: BMJ Open. 2025 Dec 22;15(12):e107063. doi: 10.1136/bmjopen-2025-107063 (PMC12730758; doi:10.1136/bmjopen-2025-107063)
Supplement: Supplementary file 3 [file bmjopen-2025-107063.reviewer_comments.pdf]

PEER REVIEW HISTORY

BMJ Open publishes all reviews undertaken for accepted manuscripts. Reviewers are asked to complete a checklist review form and are provided with free text boxes to elaborate on their assessment. These free text comments are reproduced below.

ARTICLE DETAILS

Title (Provisional)

Risk factors for cancer among the working population in China: a cross-sectional analysis of baseline data from the WECAN project

Authors

Sun, Kaige; Wang, Baohua; He, Feng J; Zhang, Puhong; Wang, Ning; Li, Yuan; Luo, Rong; Wang, Changqiong; Yi, Guangming; Yang, Ting; Zhou, Lishu; Zhou, Juan; Zhang, Guohong; Wu, Jing

---

VERSION 1 - REVIEW

**Reviewer**	**1**
**Name**	**Lynge, Elsebeth**
**Affiliation**	**Copenhagen University, Department of Public Health**
**Date**	**10-Aug-2025**
**COI**	**Done**

This is a survey of cancer related exposure factors in a small population of 841 Chinese workers from 51 factories in 3 cities. It is justified to report these data in the international literature. Mostly as a reference for comparisons internationally or over time. The most interesting part of the paper is the comparison of findings from this study with findings from previous surveys in China. I could have hoped for a comprehensive table with these comparisons; and if all "bad" prevalences had increased over time then some discussion as to why. Also some discussion about men versus women would have been interesting. What is the possible consequences of the consistently higher "bad" prevalences in men than in women; a very elderly population with a very uneven sex-distribution.

**Reviewer**	**2**
**Name**	**Abdelaziz, Enas Mahrous**
**Affiliation**	**Jouf University, Psychiatric Mental Health Nursing**
**Date**	**06-Sep-2025**

COI **None**

A few areas would benefit from refinement:

- The manuscript addresses a pertinent public health concern by investigating the prevalence of cancer risk factors within China's working population, utilizing baseline data derived from the WECAN project. The study is appropriately designed and contributes valuable epidemiological evidence that may guide the formulation of workplace-oriented cancer prevention strategies. Using of a multicenter sample encompassing three geographically diverse regions and the application of standardized data collection procedures conducted by trained personnel, both of which enhance the rigor and credibility of the findings.

A few areas would benefit from refinement:

-The study provides valuable and detailed prevalence estimates, but the discussion would be even stronger with a clearer emphasis on the practical takeaways for workplace interventions. Highlighting how the findings could guide occupational health policies or shape targeted prevention strategies would make the work more impactful and relevant for real-world application.

- The manuscript presents a comprehensive set of descriptive and multivariable analyses. The paper would be strengthened by a clearer discussion on whether these results represent true underlying disparities or are potentially attributable to sample size constraints or other study limitations.

- While the study includes data from three cities, its generalizability to the wider working population in China remains uncertain. Providing a more in-depth discussion of representativeness, possible sectoral biases (e.g., heavy industry compared to other types of workplaces).

- The manuscript appropriately acknowledges the possibility of recall and reporting bias. To strengthen the discussion, it would be helpful for the authors to elaborate on how these biases may have influenced specific outcomes, such as alcohol consumption, dietary patterns, and physical activity. Highlighting the value of incorporating objective measures in future studies could also provide useful direction for advancing this line of research."

- The results section offers a wealth of valuable data, though its density may make it challenging for readers to navigate. The addition of summary tables or clear visualizations that distill key risk factor patterns

across demographic and regional subgroups would enhance readability and support more practical interpretation by clinicians and policymakers."

VERSION 1 - AUTHOR RESPONSE

Response to Reviewer #1

Dr. Elsebeth Lynge, Copenhagen University

This is a survey of cancer related exposure factors in a small population of 841 Chinese workers from 51 factories in 3 cities. It is justified to report these data in the international literature. Mostly as a reference for comparisons internationally or over time. The most interesting part of the paper is the comparison of findings from this study with findings from previous surveys in China.

Comment 1:
I could have hoped for a comprehensive table with these comparisons; and if all "bad" prevalences had increased over time then some discussion as to why.

Response:
We sincerely thank the reviewer for this valuable suggestion. We have reorganized the discussion and summarized the comparisons with previous occupational cohort studies and the general adult population in China in **Supplementary Table S4**, providing a clearer and more intuitive presentation of sample characteristics and observed prevalences. We also expanded the discussion to address potential reasons for the observed differences, including the predominance of male employees, specific occupational characteristics, social or workplace environments that may promote unhealthy behaviours, as well as variations in survey timing, regional culture, economic conditions, and health awareness.

Modification in manuscript:
The revised discussion can be found in the second paragraph of the Discussion section.

Comment 2:
Also some discussion about men versus women would have been interesting. What is the possible consequences of the consistently higher "bad" prevalences in men than in women; a very elderly population with a very uneven sex-distribution.

Response:
We appreciate the reviewer's useful suggestion. In response, we have expanded the discussion on sex differences in risk factor exposure. Our findings indicate that men consistently exhibit higher exposure levels across lifestyle-related, biological, and occupational factors, which may partly explain their higher incidence and mortality of several chronic diseases and cancers. These patterns likely reflect gender-related differences in occupational roles, health behaviours, and risk awareness, with men more often engaged in physically demanding or hazardous work and less likely to adopt protective lifestyles or seek preventive care. This persistent imbalance may partially explain shorter life expectancy in men compared to women. We have integrated this discussion into the manuscript to better contextualize the implications of sex differences in risk factors.

Modification in manuscript:
The revised discussion can be found in the fourth paragraph of the Discussion section.

Response to Reviewer #2

Dr. Enas Mahrous Abdelaziz, Jouf University, Cairo University Faculty of Nursing

Comment 1:
The study provides valuable and detailed prevalence estimates, but the discussion would be even stronger with a clearer emphasis on the practical issues for workplace interventions. Highlighting how the findings could guide occupational health policies or shape targeted prevention strategies would make the work more impactful and relevant for real-world application.

Response:
We appreciate the reviewer's suggestion to emphasise practical implications for workplace interventions. In response, we have expanded the discussion to highlight how our findings can inform targeted occupational health strategies. Specifically, interventions should address both sex and educational disparities: men, who consistently show higher exposure to lifestyle-related, biological, and occupational risk factors, may benefit from early and sustained interventions such as promoting protective equipment use, regular health screening, and healthy behaviour programmes during their working years. Employees with lower educational levels, who exhibit higher rates of smoking, central obesity, inadequate biological monitoring, and exposure to cooking-related household air pollution, should be prioritised through accessible measures such as plain-language health education, on-site screening and counselling, and incentive-based lifestyle programmes. Furthermore, given the marked regional differences in risk factor patterns, workplace interventions should be tailored to local contexts—for example, betel quid cessation or harmless treatment policy in Xiangtan, tobacco and alcohol reduction programmes in Nanchong, and obesity management and enhanced infection screening in Wuhai. These additions strengthen the discussion by linking our prevalence estimates directly to actionable, evidence-based workplace interventions and policy recommendations.

Modification in manuscript:
These supplementary discussions are detailed in the fourth and fifth paragraph of the Discussion section.

Comment 2:
The manuscript presents a comprehensive set of descriptive and multivariable analyses. The paper would be strengthened by a clearer discussion on whether these results represent true underlying disparities or are potentially attributable to sample size constraints or other study limitations.

Response:
We thank the reviewer for this valuable comment. We acknowledge that some of the observed differences in cancer-related risk factors may be influenced by the sample size and study design. The WECAN study was originally designed as an intervention trial, with the Healthy Lifestyle Index Score (HLIS) as the primary outcome, and the sample size was calculated accordingly to detect HLIS changes between intervention and control groups. Consequently, the power for robust estimation of cancer risk factor prevalences, particularly for those with low prevalences, may be limited. We have clarified this limitation in the manuscript to ensure readers can interpret the results with appropriate caution.

Modification in manuscript:

This clarification has been added to the second-to-last and third-to-last paragraphs of the Discussion section.

Comment 3:
While the study includes data from three cities, its generalizability to the wider working population in China remains uncertain. Providing a more in-depth discussion of representativeness, possible sectoral biases (e.g., heavy industry compared to other types of workplaces).

Response:
We appreciate the reviewer's comment regarding generalizability. Although participants were recruited from 15 workplaces across three cities, covering the energy and chemical industry, steel manufacturing, and public utilities and environmental protection to include diverse occupational environments, the sampling was not designed as a nationally representative survey, which may limit the generalizability of findings to all working populations nationwide.

Modification in manuscript:

This discussion has been added to the second-to-last paragraph of the Discussion section.

Comment 4:
The manuscript appropriately acknowledges the possibility of recall and reporting bias. To strengthen the discussion, it would be helpful for the authors to elaborate on how these biases may have influenced specific

outcomes, such as alcohol consumption, dietary patterns, and physical activity. Highlighting the value of incorporating objective measures in future studies could also provide useful direction for advancing this line of research.

Response:
We thank the reviewer for this suggestion. We have expanded discussion to clarify how information and measurement biases may have affected specific outcomes. For example, alcohol intake and family history of cancer tend to be under-reported, while fruit and vegetable intake and physical activity may be over-reported. In this study, several measures were implemented to reduce bias during data collection, such as standardized training, private interviews, quality control via audio recording and logical verification in the mobile Electronic Data Capture (mEDC) system, onsite body measurements, and reference to routine health examination reports. These procedures may serve as a reference for future studies, although currently no better methods are available to further minimize such biases.

Modification in manuscript:

Relevant modifications and additional discussion can be found in the third paragraph of the Data Collection subsection in the Methods section and the final paragraph of the Discussion.

Comment 5:
The results section offers a wealth of valuable data, though its density may make it challenging for readers to navigate. The addition of summary tables or clear visualizations that distill key risk factor patterns across demographic and regional subgroups would enhance readability and support more practical interpretation by clinicians and policymakers.

Response:
We sincerely appreciate the reviewer's thoughtful and constructive comment. Given the large number of cancer risk factors examined in this study, we carefully explored several presentation formats, including vertically arranged tables and forest plots. However, these approaches did not substantially improve readability due to the volume of information. To enhance clarity while maintaining comprehensiveness, we now present the results for the composite variable "unhealthy diet" in the main text (Table 3), and provide detailed findings for individual dietary components in Supplementary Table S1. We hope this revision will make the results easier to follow for readers, clinicians, and policymakers.

We sincerely appreciate the constructive comments and suggestions from the editor and reviewers, which have helped us improve the manuscript. We hope that the revisions have adequately addressed all concerns.

VERSION 2 - REVIEW

**Reviewer**	**2**
**Name**	**Abdelaziz, Enas Mahrous**
**Affiliation**	**Jouf University, Psychiatric Mental Health Nursing**
**Date**	**05-Nov-2025**
**COI**	

Thank you for your invitation to review the manuscript "Risk factors for cancer among the working population in China: a cross-sectional analysis of baseline data from the WECAN project"

- This is a well-structured and timely study exploring the prevalence of cancer-related risk factors among China's working population. The manuscript is clearly written, methodologically sound, and

relevant for policy and workplace health promotion. However ,some aspects require clarification and strengthening to improve scientific rigor and interpretability

- -While the cross-sectional design using WECAN baseline data is appropriate, the authors should discuss more explicitly sampling framework selected workplaces as it affects representativeness at the national level.

- -Several variables are based on self-reported information . The authors should more address the potential impact social desirability bias in the Limitations section.

- The explanation of the multivariable logistic regression models is sufficient; however, the authors should provide a clarification for covariates included in the analysis.

- The use of multiple comparisons across many risk factors increases the chance of type I error; thus its important to adjusted p-values.

- the Discussion is more narrative but it needs to add more interpretations for the results

- The regional differences are described well but could be better linked to known socio-economic, cultural, or policy factors (e.g., tobacco control enforcement, dietary habits, or vaccination access.

- Tables 2 and 3 are long it may need some subheadings and rounding of percentages for readability.

- Ensure consistent terminology—for example, "central obesity" vs "abdominal obesity."

VERSION 2 - AUTHOR RESPONSE

Response to Reviewer #2

Dr. Enas Mahrous Abdelaziz, Jouf University, Cairo University Faculty of Nursing

Comment 1:

• -While the cross-sectional design using WECAN baseline data is appropriate, the authors should discuss more explicitly sampling framework selected workplaces as it affects representativeness at the national level.

Response:
Thank you for this helpful comment. We agree that the sampling framework and its implications for national representativeness should be described more explicitly. In response, we have made the following revisions:

1. **Methods section – Study design, setting and participants: clarification of workplace recruitment procedures:**

   We added a detailed description of how workplaces were identified and selected through recommendations by local CDCs followed by on-site or remote feasibility assessments. We

also clarified the inclusion criteria for participating workplaces, and noted that no additional exclusion criteria were applied.

Revised text (Methods):

"Inclusion criteria for workplaces were: (1) at least 100 full-time employees; (2) an average turnover rate <20% in the past three years; (3) willingness of the employer to participate and support project implementation; and (4) routine provision of employee health examinations. To ensure coverage of both white-collar (office-based) and blue-collar (production-based) workers, workplaces with a mix of physically demanding and non-physical occupations—such as those in the manufacturing sector—were prioritised. No additional exclusion criteria were applied. Workplaces were recruited through local Centres for Disease Control and Prevention (CDCs), which recommended eligible workplaces to the project team. The team subsequently conducted preliminary screening through on-site or remote assessments."

2. **Discussion section – acknowledgment of representativeness limitations:** We revised the Discussion to explicitly highlight that the sampling was not probability-based and that the participating workplaces therefore do not constitute a nationally representative sample. Revised text (Discussion):

"Participants were recruited from 15 workplaces across three cities, covering the energy and chemical industry, steel manufacturing, and public utilities and environmental protection sectors to include diverse occupational environments. The sampling framework was based on workplace recommendations from local CDCs followed by feasibility assessments, rather than probability-based sampling. As such, the participating workplaces reflect characteristics of the recruited workplaces rather than forming a nationally representative sample of all Chinese workplaces."

Modification in manuscript:
We have updated the second paragraph of the Study design, setting and participants section in the Methods and the second-to-last paragraph of the Discussion to address the reviewer's comment.

Comment 2:

• -Several variables are based on self-reported information. The authors should more address the potential impact social desirability bias in the Limitations section.

Response:
We thank the reviewer for highlighting the potential impact of social desirability bias. In response, we have revised the manuscript to explicitly address this issue. In the Discussion, we clarified that self-reported variables, including smoking, alcohol consumption, diet, physical activity, certain disease history, and family history of cancer, may be affected by recall and reporting biases, including social desirability bias, which could lead to under- or overestimation of the true prevalence of these lifestyle and health-related factors. Additionally, in the Strengths and Limitations section, we added a concise statement acknowledging that all self-reported lifestyle and health-related variables in our study may be subject to recall, reporting, and social desirability biases.

Revised text (Discussion):
"Finally, although several measures were implemented during data collection, self-reported variables may still be affected by recall and reporting biases, including social desirability bias. Specifically, participants may under-report unhealthy behaviours, such as smoking, alcohol consumption, certain disease history, and family history of cancer, and over-report healthy behaviours, such as fruit and vegetable intake and physical activity. These biases may lead to under- or overestimation of the true prevalence of the investigated lifestyle and health-related risk factors."

Revised text (Strengths and limitations):
"Third, several lifestyle and health-related variables, including smoking, alcohol consumption, diet, physical

activity, disease history, and family history of cancer, were self-reported and may be subject to recall, reporting, and social desirability biases."

Modification in manuscript:
The changes are reflected in the last paragraph of the Discussion and the last point of the Strengths and Limitations section.

Comment 3:

• The explanation of the multivariable logistic regression models is sufficient; however, the authors should provide a clarification for covariates included in the analysis.

Response:
Thank you for this helpful comment. We have clarified the covariates included in the multivariable logistic regression models. Specifically, sex, age group, education level, and marital status are social-demographic characteristics. The classification and description of the covariate work type are already provided in the second paragraph of the Study design, setting and participants section in the Methods. In addition, we have added a definition for the covariate family history of cancer at the end of the Definitions of Key Variables section in the Methods. It is defined as: *"Having at least one first-degree relative (parents, full siblings, or children) or second-degree relative (grandparents, uncles, or aunts) who had ever been diagnosed with any of the specified cancers."*

Modification in manuscript:

The definition of family history of cancer has been added at the end of the Definitions of Key Variables section in the Methods.

Comment 4:

• The use of multiple comparisons across many risk factors increases the chance of type I error; thus, it's important to adjusted p-values.

Response:
We appreciate the reviewer's insightful comment regarding the increased risk of type I error due to multiple comparisons across many cancer risk factors. In response, we have applied the Benjamini–Hochberg false discovery rate (FDR) correction to the p-values from the multivariable logistic regression analyses. We have also updated the Statistical Analysis section to describe this adjustment. In Table 3, statistically significant results after FDR correction are indicated with an asterisk (*).

Modification in manuscript:
Statistical Analysis section in the Methods and Table 3.

Comment 5:

• the Discussion is more narrative but it needs to add more interpretations for the results

Response:
We thank the reviewer for the comment. We have revised the Discussion section throughout, adding more interpretations of the findings and providing potential explanations for the observed patterns in several paragraphs.

Modification in manuscript:
Discussion section.

Comment 6:

• The regional differences are described well but could be better linked to known socio-economic, cultural, or policy factors (e.g., tobacco control enforcement, dietary habits, or vaccination access.)

Response:
We thank the reviewer for the suggestion. We have revised the Discussion to better link regional differences in risk factor prevalence to known socio-cultural and policy contexts. Specifically, in Xiangtan, longstanding cultural norms and recognition of betel-quid chewing as intangible cultural heritage may limit public health interventions. In Nanchong, local production and consumption patterns of tobacco and alcohol contribute to a pervasive "smoking-and-drinking culture." In Wuhai, the high prevalence of central obesity (50.0%) likely reflects colder-climate lifestyles, including lower physical activity and higher energy-dense food intake. The predominance of small private enterprises and lower economic levels may further limit health awareness and access to preventive services, reflected in low screening rates for HBsAg and HP. These changes are reflected in the revised Discussion section.

Revised text (Discussion):
"In Xiangtan, the high prevalence of betel-quid chewing (55.0%) reflects longstanding cultural practices; it was even inscribed in 2016 on Hunan Province's fourth batch of intangible cultural heritage, emphasizing its social significance. In Nanchong, elevated smoking (40.6%) and alcohol consumption (79.7%) are likely influenced by local cultural norms, as Sichuan Province produces nearly 40% of China's distilled spirits and ranks among the top five provinces in tobacco production, reflecting a pervasive "smoking-and-drinking culture." In Wuhai, the substantial burden of central obesity (50.0%) may be related to lifestyle and metabolic patterns common in colder climates, such as reduced outdoor physical activity and greater consumption of energy-dense foods; the predominance of small private enterprises and relatively lower economic levels may further limit employee welfare and health awareness, contributing to low screening rates for infection markers (37.5% for HBsAg and 24.6% for HP)."

Modification in manuscript:
The changes are reflected in the fifth paragraph of the Discussion.

Comment 7:

• Tables 2 and 3 are long it may need some subheadings and rounding of percentages for readability.

Response:
We thank the reviewer for the suggestion. In response, we have shortened Table 2 to improve readability, and adjusted the titles and headers of Table 3 and Table S1 to enhance clarity.

Modification in manuscript:
Table 2, Table 3 and Table S1.

Comment 8:

• Ensure consistent terminology—for example, "central obesity" vs "abdominal obesity."

Response:
We thank the reviewer for this valuable comment. We have carefully checked the manuscript for consistent terminology and made revisions throughout. For example, "Cancer-related risk factors" has been standardized to "cancer risk factors", and "obese" has been unified as "obesity".

We sincerely appreciate the constructive comments and suggestions from the reviewers, which have helped us improve the manuscript. We hope that the revisions have adequately addressed all concerns.

Thank you for your consideration.
